# High-quality genome assembly of *Aglaia odorata* reveals evolution, terpenes diversity and abundance in Meliaceae

**DOI:** 10.1186/s43897-025-00212-9

**Published:** 2026-04-09

**Authors:** Zhiyu Chen, Xingyu Yang, Tianyu Yang, Xin Yin, Danni Yang, Xuefei Yang, Yunqiang Yang, Yongping Yang

**Affiliations:** 1 CAS Key Laboratory of Tropical Plant Resources and Sustainable Use, Xishuangbanna Tropical Botanical Garden, Chinese Academy of Sciences, Kunming, 650223 China; 2 Plant Germplasm and Genomics Center, Kunming Institute of Botany, Chinese Academy of Sciences, Kunming, 650201 China; 3 University of Chinese Academy of Sciences, Beijing, 100049 China; 4 Key Laboratory of Economic Plants and Biotechnology, Kunming Institute of Botany, Chinese Academy of Sciences, Kunming, Yunnan 650201 China

**Keywords:** Meliaceae, Whole-genome triplication, Subgenome dominance, Terpene biosynthesis, Transcription regulation

## Abstract

Meliaceae species are known for producing diverse metabolites, yet the role of whole-genome duplication (WGD) in shaping this diversity remains unclear. In this study, we analyzed volatile metabolites in *Azadirachta indica*, *Toona ciliata* and *Aglaia odorata*, finding terpenes to be most abundant in *A. odorata*. A high-quality genome assembly of *A. odorata* revealed lineage-specific whole-genome triplication (WGT), the first reported in Meliaceae. Comparative genomics indicated extensive chromosomal rearrangements, biased gene retention, gene expression, and long terminal repeat (LTR) insertions during the evolution of subgenomes in Meliaceae, with *A. odorata* showing subgenome dominance. Despite a reduction in terpene synthase (TPS) gene copies, the specifically highly expression of *AodTPS25, AodTPS21, AodTPS24* and *AodTPS26* in *A. odorata* could be crucial for its terpene abundance and diversity through transcriptomics and enzyme assays. Moreover, the ethylene response factor AodERF27 was found to activate *AodTPS26*, linking transcriptional regulation to terpene diversity. These findings uncovered the genomic and molecular mechanisms in Meliaceae.

**Supplementary Information:**

The online version contains supplementary material available at 10.1186/s43897-025-00212-9.

## Core

We present a high-quality genome assembly of *Aglaia odorata* and integrate comparative genomic, transcriptomic, metabolomic, and molecular assays to uncover the mechanisms driving elevated terpene diversity. Our study highlights how WGD/WGT events influence gene expression and metabolite diversity, offering new insights into the gene architecture underlying plant metabolite diversity.

## Gene and accession numbers

The genome data generated from this study have been deposited in the National Genomics Data Center under accession number CRA020431. The genome assembly and annotation could be obtained from Figshare (10.6084/m9.figshare.27794091.v1).

## Introduction

Meliaceae species are widely distributed across tropical and subtropical regions, playing a vital role in ecosystems because of their diverse morphologies and growth characteristics. Notably, these species are rich in terpenes compounds with distinct structures and bioactivity that contribute to plants’ scent, medicinal properties, and economic value. For example, *Azadirachta indica* produces azadirachtin-rich essential oil used as natural insecticides (Khan 2021). *Toona sinensis*, known as the "tree vegetable", is valued for its unique flavor (Wang et al. 2014), while *Toona ciliata*, a relative species of *T. sinensis*, is prized for its natural and pleasant fragrant wood (Duan et al. 2015). *Aglaia odorata* serves as a fragrance resource in perfumery and cosmetics (Weyerstahl et al. 1999), is commonly used as traditional medicine to treat injuries, and heart conditions (Kato-Noguchi et al. 2016). Terpene composition across Meliaceae species underlies differences in ecological adaption, defense, and human application (Greger 2021, Tundis et al. 2014). However, the biosynthetic diversity of these terpenes remains insufficiently characterized, limiting their exploration and utilization.

The whole-genome duplication (WGD), numerous genes are simultaneously duplicates with the addition of extra sets of genomes, providing crucial genetic material for evolutionary innovation (Soltis and Soltis 2016, Cheng et al. 2018) and driving species diversity and metabolic complexity (Moriyama and Koshiba-Takeuchi 2018). Meliaceae, a diverse family within eudicots, has a complex evolutionary history. In addition to the ancient whole-genome triplication (WGT) event shared by core eudicots, lineage-specific WGD events have been reported in *T. ciliata* (Wang et al. 2022) and *T. sinensis* (Ji et al. 2021), but not in *A. indica* (Du et al. 2022). These independent WGD events likely contributed to genetic divergence and enhanced adaptive potential in Meliaceae species. However, their effects on metabolic diversity remain largely unexplored. Following WGD, the genome often undergoes diploidization, shaping it into a quasi-diploid state through processes such as chromosomal rearrangement, biased gene retention or loss, gene sub/neofunctionalization, gene expression reprogramming and transposable element (TE) activation (Qiao et al. 2019, Mandakova and Lysak 2018). These evolutionary events significantly influence species’ adaptability, morphological traits, and metabolic pathways (Cheng et al. 2014, Edger et al. 2015, Hofberger et al. 2013).

During diploidization, the asymmetrical loss of duplicated genes shapes phenotypic traits and metabolic pathways, driving species divergence and diversity (Deb et al. 2023). While the key roles of WGD events and subsequent diploidization are widely recognized (Wu et al. 2020, Qiao et al. 2022), the mechanisms by which gene loss promotes species diversity remain underexplored. In Meliaceae, the evolutionary trajectories of gene families following WGD are particular unclear. Given the frequent WGD events in this family, Meliaceae provides a valuable system for investigating how genome duplication influences gene families, metabolic diversification, and species adaptation.

In this study, we generated a high-quality genome of *A. odorata* and systematically compared the volatile metabolite profiles across three Meliaceae species (*A. indica, T. ciliata and A. odorata*), revealing that *A. odorata* produces the highest levels of terpenes. Genomic and comparative genome analysis revealed a lineage-specific WGT event in *A. odorata*, the first reported in Meliaceae. Surprisingly, despite its high terpene level, *A. odorata* showed a contraction in terpene synthase (TPS) gene copies after WGT. To investigate this paradox, we examined *AodTPS*s gene expression, function and promoter regulation using transcriptomics profiling, enzymatic activity assays, and promoter activation studies. This work sheds light on the genomic and regulatory mechanisms shaping terpene diversity in Meliaceae.

## Results

### Volatile compounds in three Meliaceae species

**Fig. 1:**
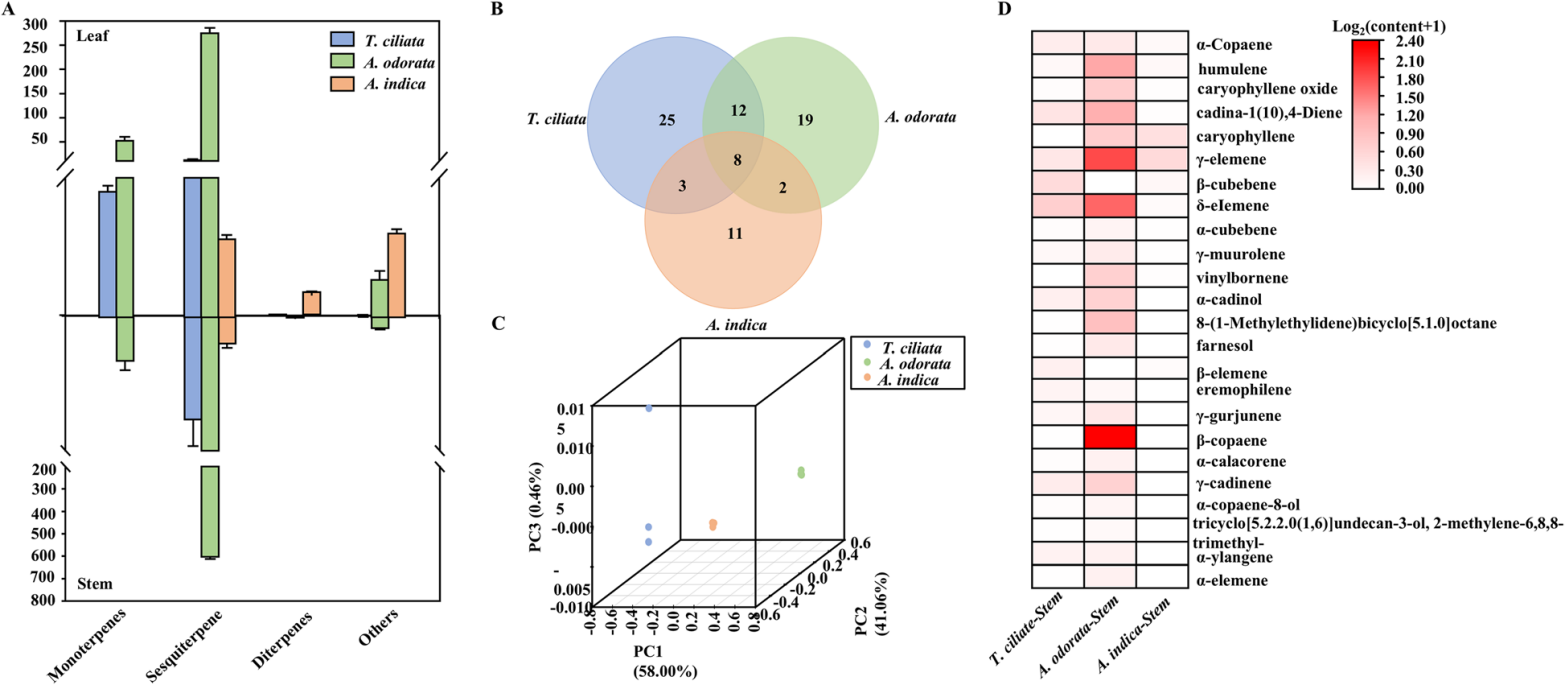
Essential oils in three Meliaceae species. **A** Histogram showing the content of monoterpenes, sesquiterpenes, diterpenes, and other compounds in the leaves and stems of *A. indica*, *T. ciliata*, and *A. odorata*. The x axis presents conpound types, the y axis represents content level (ng/g). **B** Venn diagram of essential oils categories shared among stems of three species. **C** PCA of essential oil profiles from stems of *A. indica*, *T. ciliata* and *A. odorata*. Axes represent relative distance; dot colors represent different samples. **D** Heat maps of mainly essential oil content in the stems of *A. indica*, *T. ciliata* and *A. odorata*

To explore interspecific differences in volatile compounds among Meliaceae species, essential oils were extracted from the leaves and stems of *A. indica, T. ciliata*, *and A. odorata*, which were collected from a common garden experiment. *A. odorata* exhibited significantly higher volatile concentration, 329.0 μg/g in leaves and 614.8 μg/g in stems, approximately 50 and 107 times higher than *A. indica* and 19 and 28 times higher than *T. ciliata*, respectively (Fig. 1A). Compound profiling identified 31, 27, and 38 volatile compounds in the leaves, and 24, 48, and 41 in the stems of *A. indica, T. ciliata*, *and A. odorata*, respectively (Fig. 1B; Fig. S1). Principal component analysis (PCA) clearly separated the species into distinct clusters based on volatile profiles in both leaves and stems (Fig. 1C; Fig. S2), reflecting strong metabolic divergence among the species.

In *A. odorata* leaves, sesquiterpenes and monoterpenes dominated (Fig. 1A), with caryophyllene and β-cubebene comprising 89.6% of the total volatiles, 81 and 17 times higher than in *A. indica* and *T. ciliata*, respectively. In contrast, *A. indica* primarily produced sesquiterpenes such as γ-elemene and phyrol, along with nitrogenous compounds, while *T. ciliata* characterized sesquiterpenes such as bicyclogermacrene, caryophyllene, and hydrocarbon artemisiatriene. A Similar pattern was observed in stem tissues (Fig. S3). *A. odorata* stems were rich in terpenes such as copaene, caryophyllene, and γ-elemene, accounting for 98.4% of total content, 105 and 28 times higher than in *A. indica* and *T. ciliata*, respectively (Fig. 1D). Species-specific compounds further highlighted metabolic uniqueness: ꞵ-patchoulene was exclusive to *A. odorata*, whereas α- neoclovene and humulen-(V1) were specific to *A. indica* and *T. ciliata*, respectively. These variations in compound composition and ratios suggest the biosynthetic pathways of volatile metabolites have diverged significantly across these Meliaceae species, potential contributing to their distinct ecological roles and aromas.

### Genome sequencing, assembly, and annotation of *A. odorata*

To elucidate the genetic basis underlying differences in volatile compound production and address the lack of a reference genome for *A. odorata*, we performed genome sequencing using Illumina, Nanopore, and PacBio HiFi platform for *A. odorata* (Fig. S4). K-mer analysis estimated the genome size at 471.84 Mb with high heterozygosity (approximately 0.98 %) (Fig. S5). In total, we generated 96 Gb (203× coverage) of PacBio HiFi, 56 Gb (118× coverage) of Nanopore, and 31 Gb (65× coverage) of Illumina sequencing data for assembly (Table S1). After the primary assembly and polishing, *A. odorata* haplotypes 1 (Hap1) and 2 (Hap2) were assembled to sizes of 499.90 Mb (contig N50: 10.70 Mb) and 456.14 Mb (contig N50: 10.46 Mb), respectively (Table S2).

**Fig. 2:**
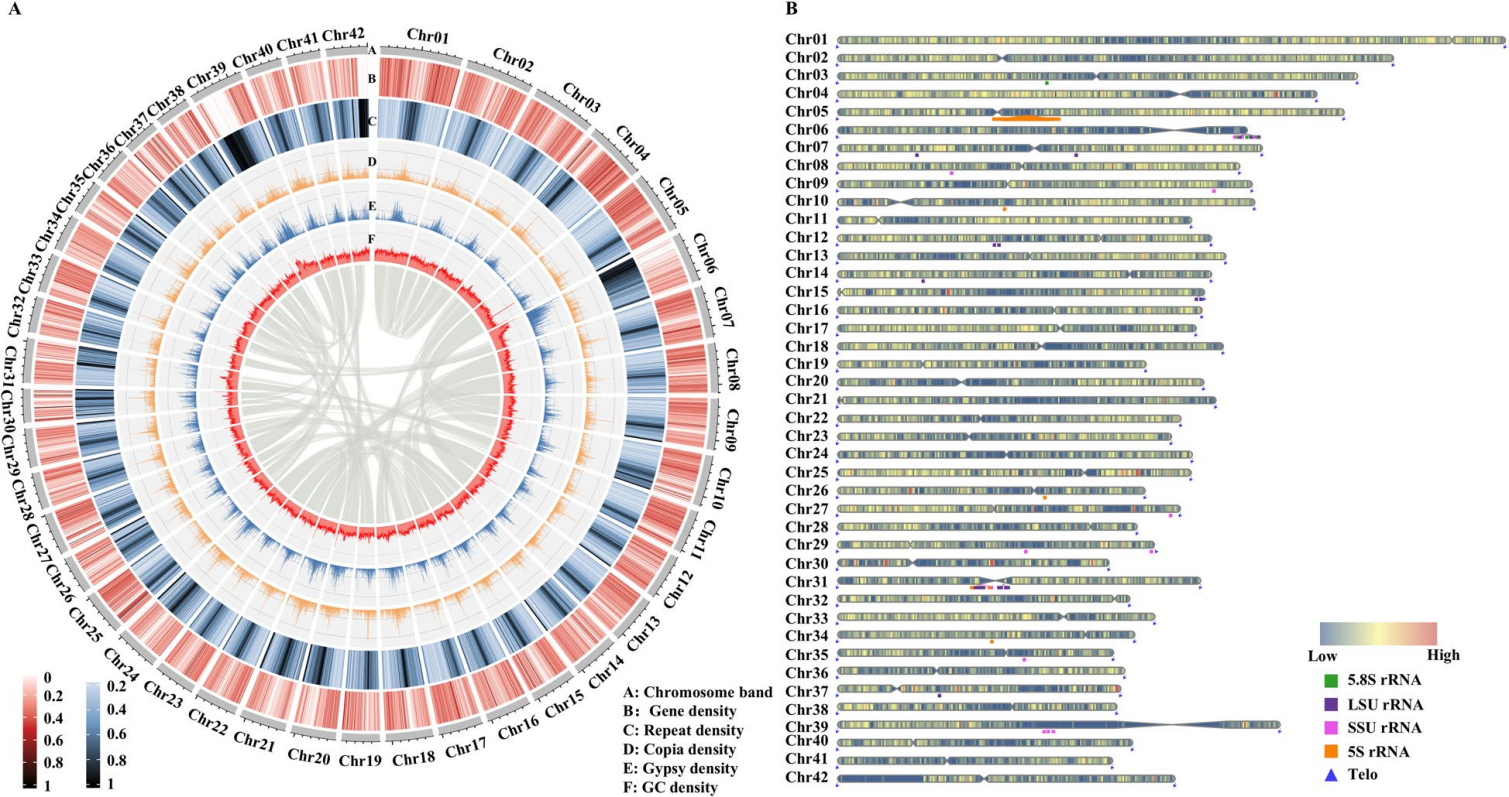
Genome sequencing of *A. odorata*. **A** Genomic feature overview of *A. odorata* with Chr01-Chr42 presenting pseudochromosomes. Inner links denote collinear blocks. **B** Telomere and centromere detection map

Using 55.29 Gb (117× coverage) of Illumina Hi-C sequencing data, the assemblies were anchored to 42 pseudochromosomes (Chr01-Chr42), ranging in from 7.16 to 19.93 Mb (Table S3 and Fig. S6), consistent with chromosome counts reported in the CDBB database. After redundancy removal and gap filling, near telomere-to-telomere (T2T) assemblies were achieved, excluding a telomere missed on chromosome 6, with final genome sizes of 462.31 Mb for Hap1 and 448.91 Mb for Hap2 (Fig. 2A and B; Table 1). Assembly completeness was confirmed by BUSCO scores of 98.3% (Hap1) and 98.1% (Hap2) (Table S4), indicating high quality of *A. odorata* genome.

**Table 1:** Statistics of genome assembly and annotation of *A. odorata*

	Hap1	Hap2
Genome size (Mb)	462.31	448.91
Contig N50 (Mb)	12.10	10.43
Pseudochromosome number	42	42
Complete BUSCOs for genome (%)	98.3	98.1
Protein-coding gene number	33965	33874
Repeat sequences (Mb)	175.82	163.18
Complete BUSCOs for protein (%)	98.1	97.9

Repeat annotation identified 175.82 (38.03%) and 163.18 Mb (36.35 %) of TE in Hap1 and Hap2, respectively, with long terminal repeats (LTRs) including Copia and Gypsy being the most abundant, followed by terminal inverted repeats (TIRs) (Table S5 and Table S6). Gene annotation, based on homology, *de novo* and transciptome-based methods, identified 33,965 and 33,874 protein-coding genes in Hap1 and Hap2, respectively. Of these genes, 97.68% and 97.95% were functionally annotated through protein databases, respectively (Table S7). Additionally, 2,111 (Hap1) and 1,989 (Hap2) non-coding RNAs were predicted (Table S8). BUSCO results confirmed high completeness of annotated genes: 98.1% for Hap1 and 97.9% for Hap2 (Table S9). Given its higher BUSCO score and gene count, Hap1 was selected for downstream analyses.

### Genome evolution of Meliaceae species

**Fig. 3:**
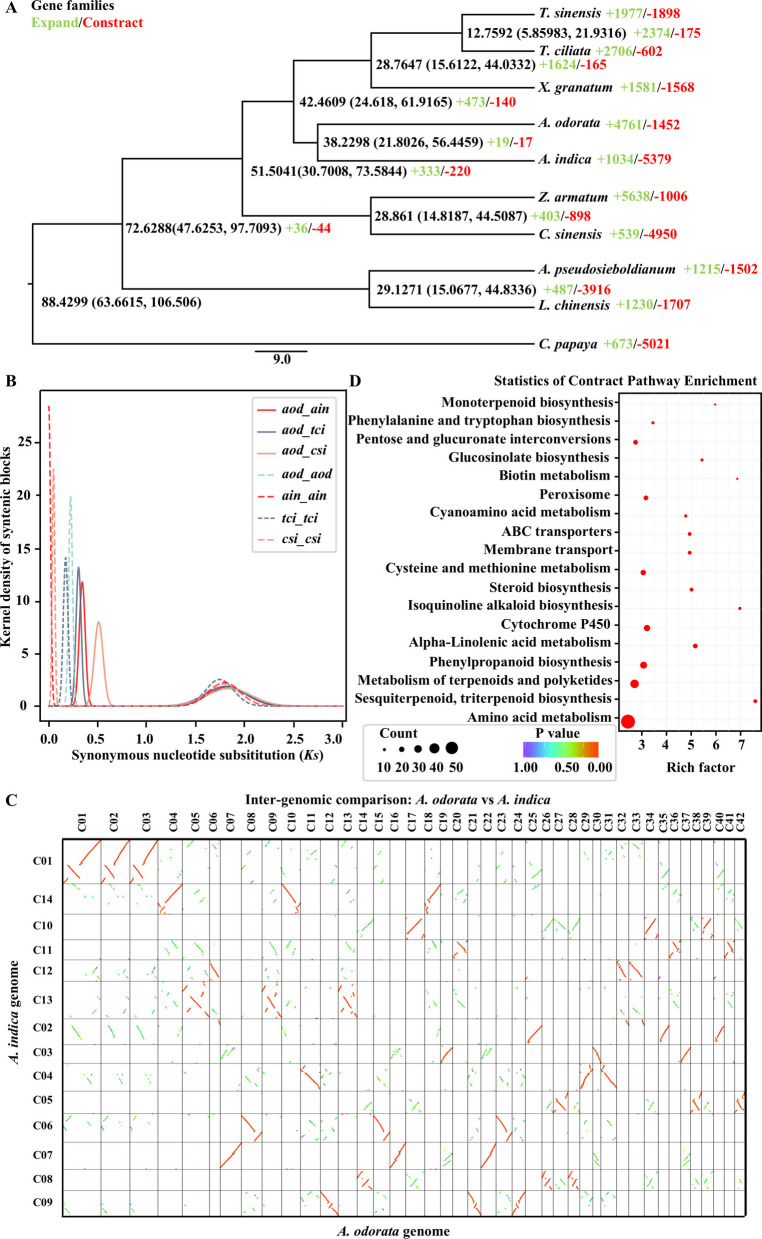
Genome evolution of *A. odorata*. **A** Phylogenetic tree based on single-copy orthologs accross 9 Sapindales species and one outgroup.Node labels indicate estimated divergence times (Mya); red and green numbers represent expanded and contracted gene families, respectively. **B** Density distribution of synonymous nucleotide substitution rates (*Ks*) in *A. indica*, *T. ciliata*, *A. odorata*, and *C. sinensis.* **C** Dot plots syntenic blocks showing 1:3 chromosomal relationship between *A. indica* and *A. odorata* genome. **D** KEGG pathway enrichment of contracted gene families in *A. odorata*

To explore the evolutionary process of Meliaceae species, a phylogenetic tree was constructed using 308 single-copy orthologous genes from nine Sapindales species, with *Carica papaya* as the outgroup. *A. odorata* clustered with *A. indica* in a single clade, namely the Melioideae subfamily of Meliaceae, with an estimated divergence time of 38 million years ago (Mya). The divergence between Melioideae and Cedreloideae (*T. ciliata*, *T. sinensis*, and *X. granatum*), was estimated to 42.46 Mya (Fig. 3A). *Ks* analysis of homologous gene pairs showed a shared *Ks* peaks (1.74–1.84) among *A. odorata*, *A. indica*, *C. sinensis*, and *T. ciliata*, consistent with the WGT event shared by core eudicots (Jiao et al. 2011). Additional signature *Ks* peaks of paralogous gene pairs at 0.22 (*A. odorata*) and 0.17 (*T. ciliata*), both lower than their orthologous peak (0.30), suggesting lineage-specific polyploidization event after speciation (Fig. 3B). Gene homology dot plot revealed a 3:1 syntenic pattern between *A. odorata* and *A. indica* (Fig. 3C), 3:1 with *A. odorata* and *C. sinensis* (Fig. S8), and 3:2 for *A. odorata* and *T. ciliata* (Fig. S9). Given that *A. indica* (Du et al. 2022) and *C. sinensis* (Wu et al. 2023) did not undergo any recent WGD event, these results support a lineage-specific WGT event in *A. odorata*, the first reported in Meliaceae. Re-sequencing data from five individual of *A. indica, T. ciliata* and *A. odorata* showed mapping rates to their reference genome exceeding 74.0%, 98.9%, and 95.9%, respectively (Table S11), indicating low intraspecific variation and suggesting that observed WGD/WGT events were species-wide, not individual-specific. Using a neutral substitution rate (*r*) of 3.86E-09, the WGT in *A. odorata* was estimated to have occurred approximately 28.49 Mya, which was after it diverged from *A. indica*. Moreover, recent bursts of LTR insertions were observed in *A. odorata* (0.4 Mya) and *T. ciliata* (2.2 Mya), but not in *A. indica* (Fig. S10).

Homology searches of pseudogenization were conducted to identify candidate pseudogenes in the Meliaceae genomes. *A. odorata* harbored the most pseudogenes among Meliaceae species (Fig. S11), implying pseudogenization may contributed to reverting its genome to a diploid-like state following WGT. Gene family analysis revealed that *A. odorata* experienced the most extensive gene expansion, likely driven by WGT. The KEGG enrichment of expanded gene families in *A. odorata* and *T. ciliata* revealed significant enrichment in environmental adaptability-related processes, particularly “plant-pathogen interaction” and “replication and repair” pathway (Fig. S12 and Fig. S13). Interestingly, despite its elevated terpene levels (Fig. 1A), *A. odorata* exhibited contraction in terpene biosynthesis-related gene family (Fig. 3D). This discrepancy suggests that enhanced terpene production in *A. odorata* may be regulated through mechanisms beyond gene copy number, such as transcriptional or enzymatic regulation.

### Biased subgenome evolution in *A. odorata*

**Fig. 4:**
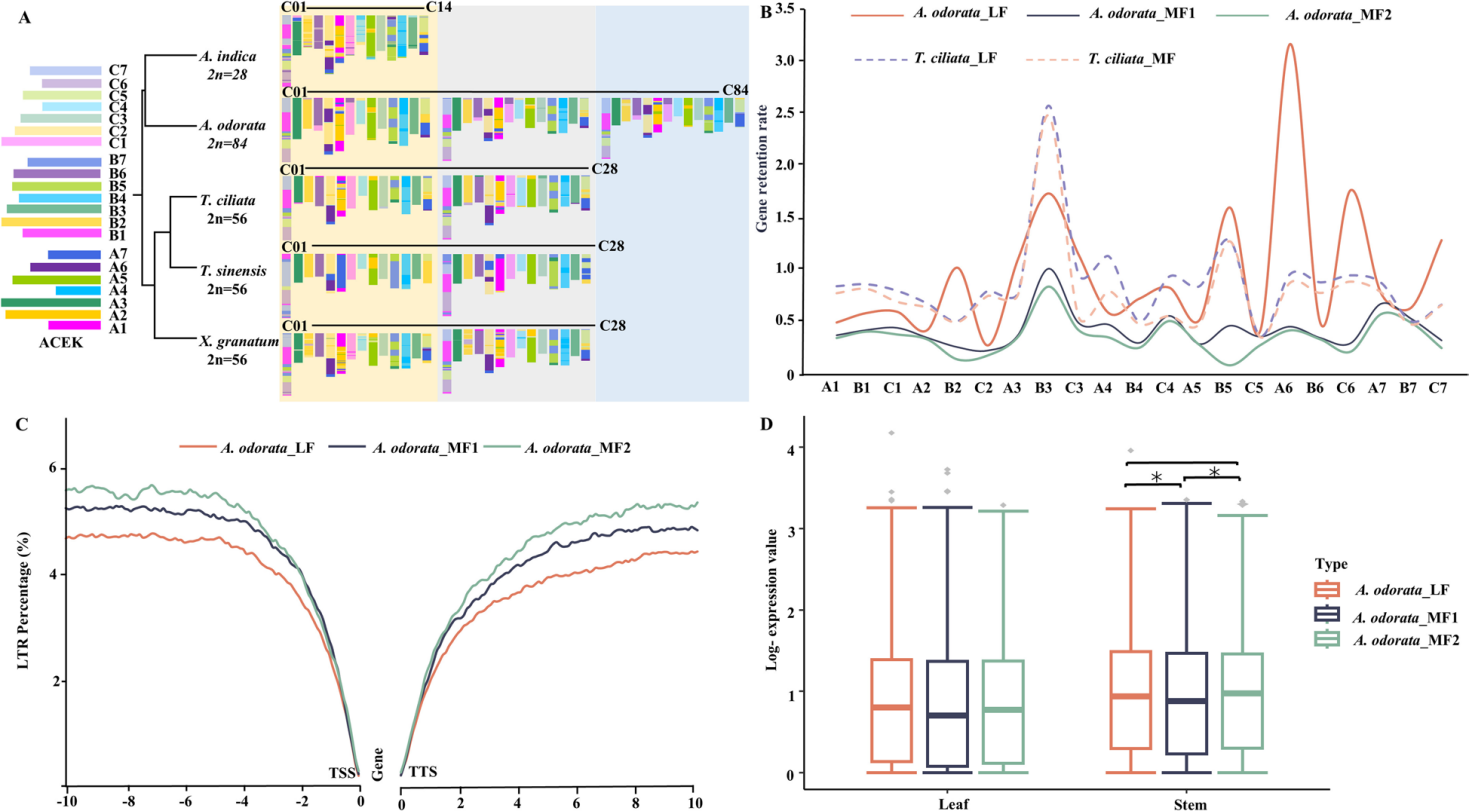
Subgenome evolution in *A. odorata*. **A** Evolutionary models of five Meliaceae species based on 21 ancestral core eudicots karyotypes (ACEK) blocks. **B** Gene retention ratios of *A. odorata* and *T. ciliata* subgenomes relative to *A. indica*, across ACEK blocks (A1-A7, B1-B7, C1-C7). The x axis presents the 21 ACEK blocks; the y axis represents the gene retention ratio. **C** LTR density within 10 kb upstream and downstream of genes in *A. odorata* subgenomes, shown in 100 bp pane. The x axis represents distance from the gene; the y axis represents the LTRs percentage. **D** Box plots showing expression levels (log-transforned) of genes from the three *A. odorata* subgenomes (LF, MF1 and MF2) in leaf and stem tissues. The x axis denotes tissue type; the y axis shows gene expression values

To investigate chromosomal rearrangements and subgenome evolution during diploidization, we reconstructed the karyotypes and subgenomes of *A. odorata*, *A. indica*, *T. ciliata*, *T. sinensis*, and *X. granatum* using the 21 ancestral core eudicots karyotypes (ACEK) chromosomes (A1-A7, B1-B7, C1-C7) as ancestral blocks (Fig. 4A and Fig. S17). *A. indica*, which did not undergo WGD or WGT, maintained 28 chromosomes (2n=28). In contrast, *T. ciliata*, *T. sinensis*, and *X. granatum* experienced a WGD event, doubling their chromosome number to 56 (2n=56) and resulting in two subgenomes, least-fractionated (LF) and max-fractionated (MF) based on gene retention. *A. odorata*, following a lineage-specific WGT event, remained 84 chromosomes (2n=84) and three subgenomes: LF, medium-fractionated (MF1), and most-fractionated (MF2) (Table S12).

Compared to the ancestral karyotype, *A. indica* exhibited both conserved and rearranged chromosomal structures. For consistency, chromosomes across species were numbered according to *A. indica*. Chromosomes C02, C04 and C10 in *A. indica* were mainly derived from ancestral blocks A3 (84.8%), B6 (91.5%) and C3 (100%) of ACEK, respectively, indicating strong conservation. In contrast, chromosome C01 of *A. indica* contained a mosaic of B1, C5, C6, and C7, reflecting extensive chromosome fissions, fusions and rearrangements. In Cedreloideae species and *A. odorata*, WGD and WGT events, respectively, led to the evolution of two (*T. ciliata*, *T. sinensis*, and *X. granatum*) or three (*A. odorata*) subgenomes. While these species remained many conserved ACEK blocks, their genomic architecture diverged notably in block composition and distribution compared to *A. indica*. For example, *T. ciliata* chromosomes C04 and C18, which correspond to *A. indica* chromosome C04, contained ACEK blocks B6, B1 and A3, suggesting that *T. ciliata* had undergone substantial structural reshuffling post-divergence (Fig. 4A). Further subgenomic variations were observed within *A. odorata*. Chromosomes C08, C22, and C36, each from different subgenomes, were primarily derived from ACEK block C4, with chromosomes C08, C22 also incorporating blocks from C2, forming complex mosaics (Fig. 4A). These results suggest that chromosomal structural variations occurred both within and between species and subgenomes after speciation and polyploid events. Such rearrangements, potentially contributed to the karyotypic diversity and evolutionary divergence observed across Meliaceae species.

To further clarify subgenomes’ evolution, we analyzed LTR insertions and gene expression across the subgenomes of three representative Meliaceae species. In *A. odorata*, the proportion (but not the absolute number) of LTR insertions within gene coding regions upstream/downstream was highest in MF2 subgenome, whereas DNA transposon content showed no such pattern (Fig. 4C, Fig. S18 and S19). In contrast, *T. ciliata* showed no significant differences in LTR or DNA insertions rates between its two subgenomes (Fig. S19 and S20), indicating that *A. odorata* experienced asymmetric LTR accumulation of subgenomes. LTR burst time also varied: ~0.4 Mya in LF and MF1, but ~0.5 Mya in MF2 (Fig. S21), supporting differential subgenome evolution. Transcriptomic analysis revealed significantly higher expression of syntenic genes in LF than in MF1 in *A. odorata* stems (Fig. 4D), indicating a gene expression bias and subgenome dominance. Together, these findings demonstrate that *A. odorata* exhibits subgenome asymmetry, characterized by biased LTR insertions and differential gene expression, reflecting divergent evolutionary trajectories among its subgenomes following the WGT event.

### Terpene biosynthesis genes

**Fig. 5:**
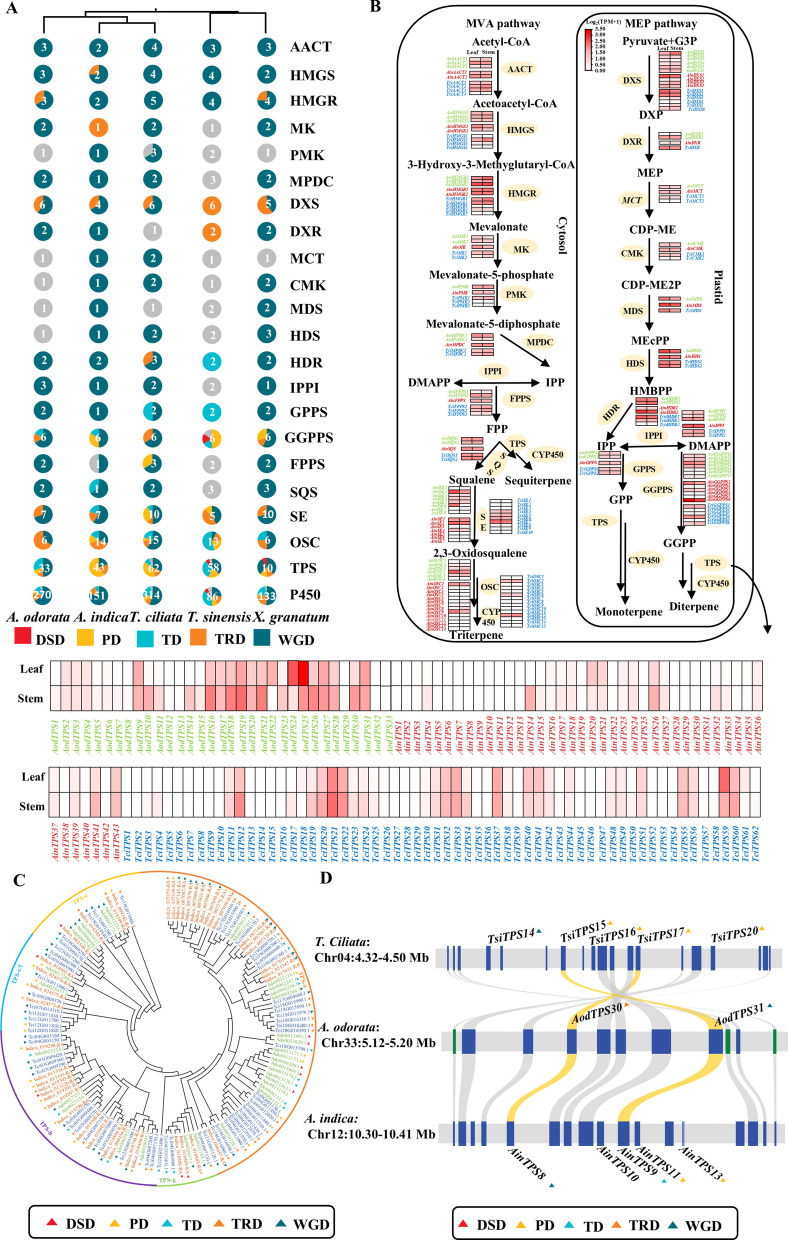
Terpene biosynthetic pathway analysis in Meliaceae species. **A** Numbers and duplication sources of terpene biosynthesis genes in five Meliaceae species. The number and color in the circle represent gene count and duplication sources, respectively. **B** Heatmaps of terpene biosynthesis gene expression in leaves and stems of three Meliaceae species. Font colors represent species: blue for *T. ciliata*, green for *A. odorata*, and red for *A. indica*. **C** Phylogenetic tree of TPS from the three Meliaceae species, cclassified into subfamilies a-g. Triangle colors represent different duplication sources. **D** Intergenomic synteny of *TPS* gene blocks in *T. ciliata* Chr04, *A. odorata* Chr33, and *A. indica* Chr12. Syntenic *TPS* gene pairs are connected by yellow lines; triangle colors represent duplication sources

To investigate the paradox of high terpene abundance despite a contraction in terpene biosynthesis-related genes in *A. odorata*, we identified all genes involved in terpene biosynthesis across five Meliaceae species. A total of 29, 50, 45, 42 and 42 upstream terpenoid biosynthesis genes were detected in *A. indica*, *T. ciliata*, *T. sinensis*, *X. granatum*, and *A. odorata*, respectively. These genes were involved in the methylerythritol phosphate (MEP) and mevalonic acid (MVA) pathways. Notably, *A. indica*, which lacks a recent WGD/WGT event, had fewer gene copies, with nearly half being singletons. In contrast, other species showed gene expansion mainly due to WGD, with some genes also arising from tandem or dispersed duplications (TRDs) (Fig. 5A and B).

To further assess the influence of WGD/WGT events on terpenoid metabolism, we performed a comparative genomics analysis of key pathway genes among three representative species: *A. indica* (no WGD/WGT), *T. ciliata* (WGD), and *A. odorata* (WGT). Genes, such as acetyl-CoA acetyltransferase gene (*AACT)*, hydroxymethylglutaryl-CoA synthase gene (*HMGS*), and isopentenyl pyrophosphate isomerase gene (*IPPI*), exhibited a clear 1:2:3 syntenic pattern across these species, indicating expansion via WGD/WGT events in *T. ciliata* and *A. odorata*. In contrast, other key genes, such as mevalonate diphosphate decarboxylase gene (*MPDC*), mevalonate kinase gene (*MK*), and 4-hydroxy-3-methylbut-2-en-1-yl diphosphate synthase gene (*HDS*), retained only one or two copies in *A. odorata*, suggesting post-WGT gene loss. Meanwhile, phosphomevalonate kinase gene (*PKM*) and mevalonate diphosphate decarboxylase gene (*MDS*) remained single copy with conserved synteny in all three species (Fig. S24, S25, and S26), highlighting selective gene retention during *A. odorata* evolution.

Interestingly, the gene copies of terpene synthase (TPS), key enzymes catalyzing terpenoid skeleton biosynthesis (Karunanithi and Zerbe 2019), varied notably among Meliaceae species. *T. ciliata* had the highest number of *TPS* genes with a count of 62, followed by *A. indica* with 43 *AinTPSs*, and *A. odorata* with 33 *AodTPSs* (Fig. 5A). Phylogenetic analysis grouped the *TPSs* into five subfamilies: *TPS*-a, *TPS*-b, *TPS*-c, *TPS*-e/f, and *TPS*-g. Subfamily-specific differences were especially prominent. The *TPS*-b subfamily, typically associated with monoterpene biosynthesis, included 14, 16, and only 5 members in *A. indica*, *T. ciliata*, and *A. odorata*, respectively, indicating a marked contraction in *A. odorata*. Similarly, the *TPS*-a subfamily contained 17, 28, and 21 members, while the *TPS*-e/f subfamilies had 4, 9, and just 2 members in the respective species (Fig. 5C). These findings suggest that despite undergoing a WGT event, *A. odorata* experienced significant gene contraction in the *TPSs* families, particularly in the *TPS*-a, *TPS*-b and *TPS*-e/f subfamilies.

To clarify the variation in *TPS* gene copy number among the three Meliaceae species, we conducted gene synteny and origin analyses. A subset of *TPS* genes: *AinTPS3*, *TciTPS59*/*TciTPS60*, and *AodTPS1*/*AodTPS2*/*AodTPS3*, exhibited conserved synteny with a clear 1:2:3 correspondence, consistent with the species’ WGD/WGT histories. However, most *TPSs* genes exhibited complex “many-to-one” syntenic relationship. For example, *AinTPS43* and *AodTPS22*, syntenic with each other, corresponding to three *TciTPS* genes *TciTPS31*, *TciTPS32*, and *TciTPS33* (Fig. S27), suggesting gene loss in *A. odorata* and lineage-specific duplications in *T. ciliata*. *TPS* gene origin analysis revealed that *AodTPSs* originated primarily from WGD and tandem duplication (TD) events, while *AinTPSs* and *TciTPSs* also exhibited prominent expansion via proximal duplication (PD). For example, *AodTPS30*, *TciTPS17* and *AinTPS8* shared strong synteny, as did *AodTPS31*, *TciTPS15* and *AinTPS9.* PD-derived genes were also found in *A. indica* (e.g., *AinTPS11*, *AinTPS13*) and in *T. ciliata* (e.g., *TciTPS14*, *TciTPS16*, and *TciTPS20*), with some (*AinTPS10*) of uncertain origin. These results indicated that TD and PD events were responsible for the expansion of *AinTPSs* and *TciTPSs* (Fig. 5D). Overall, despite undergoing a lineage-specific WGT event, *A. odorata* experienced *TPSs* gene contraction, while PD events played a major role in *TPS* expansion in *A. indica* and *T. ciliata*. These distinct evolutionary mechanisms likely underlie the diversity in *TPS* gene copy number across Meliaceae species.

### Functional characteristics of *AodTPSs*

Transcriptome analysis of leaves and stems from *A. indica*, *T. ciliata*, and *A. odorata* revealed significant differences in *TPS* gene expression. Despite having fewer *TPS* genes, *A. odorata* showed markedly higher expression, with 7 and 13 *AodTPSs* exhibiting high expression levels (TPM>15) in leaves and stems, respectively. In contrast, the majority of *TPS*s in *A. indica* (37/43) and *T. ciliata* (47/62) were either lowly expressed or not expressed at all (Fig. 5A). These finding suggest that *A. odorata* compensates for its reduced *TPS* gene copy number with elevated expression of specific *AodTPSs*, which likely contributed to its high terpene content. This indicates a shift toward regulatory enhancement rather than gene expansion in the evolution of terpene biosynthesis in *A. odorata*.

**Fig. 6:**
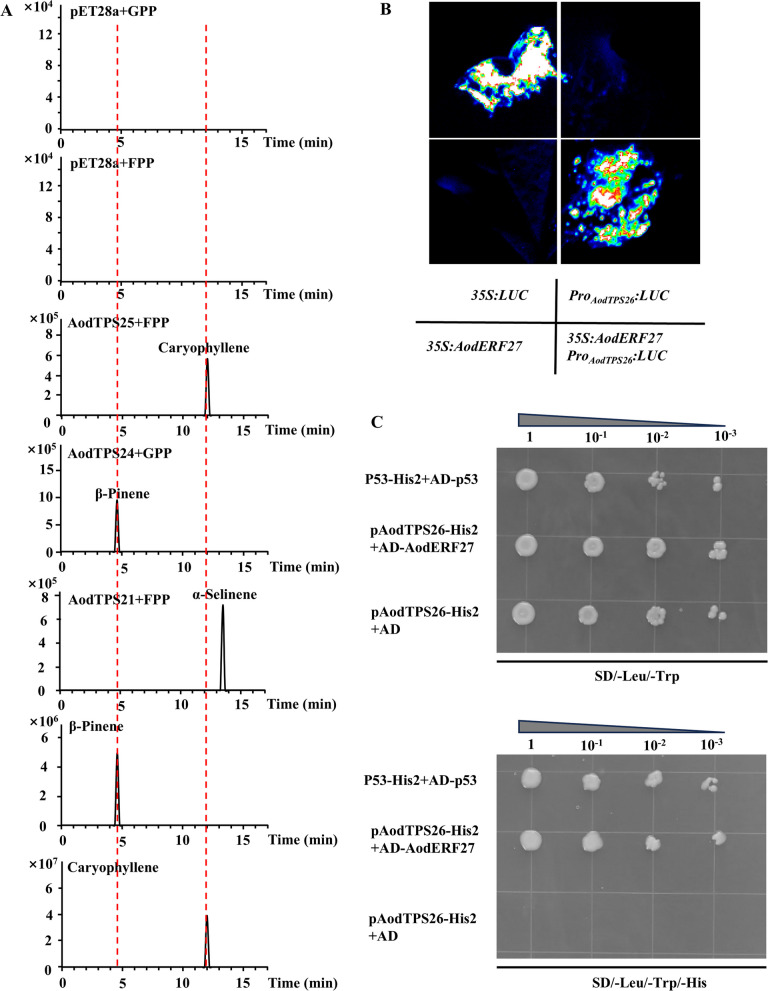
Genetic basis of volatile terpene differences in Meliaceae species. **A** GC-MS spectra showing representative compound peaks of terpene products catalyzed by purified AodTPS21, AodTPS24, and AodTPS25. The x axis represents retention time; the y axis represents relative abundance. **B** Dual-luciferase assays in *N. benthamiana* assessing *AodTPS26* promoter activity regulated by AodERF27. **C** Yeast one-hybrid assay of AodERF27 bingding to the *AodTPS26* promoter. pAodTPS26-His2+AD served as the negative control;; p53-His2+AD-p53 was used as the positive control

To elucidate the enzymatic roles of *AodTPSs* in *A. odorata*, several candidates with distinct tissue-specific expression patterns were selected for functional assays. *AodTPS25* (*Aod0033820*), a TPS-a subfamily member and the most highly expressed *AodTPSs* in leaves, was cloned, expressed in *E. coli* BL21 (DE3), and protein purified. In vitro enzymatic assays showed that it catalyzed the conversion of farnesyl pyrophosphate (FPP) into caryophyllene (Fig. 6A). AodTPS21 (Aod0015995), a species-specific TPS-a protein, predominantly expressed in stems, catalyzed the formation of α-selinene from FPP (Fig. 6A). AodTPS24 (Aod0021234), the most highly expressed *AodTPS* in both leaves and stems of *A. odorata* (Fig. S28), belongs to the TPS-b subfamily, which typically produces monoterpenes. It was functionally validated as a β-pinene synthase using geranyl pyrophosphate (GPP) as a substrate (Fig. 6A).

*AodTPS26* was identified as the most distinct and specifically expressed *AodTPS* in *A. odorata* (Fig. 5A), exhibiting high stem-specific expression and belonging to the TPS-a subfamily. In contrast, its homologs in *A. indica* (*Indica_017010*) and *T. ciliata* (*Tci19G002100*) were minimally expressed in both leaves and stems (Fig. S29). Despite these expression differences, predicted protein structures of the three orthologs revealed conserved 3D architectures (Fig. S30). Consistently, in vitro enzymatic assays demonstrated that all three enzymes catalyzed the conversion of FPP to α-farnesene (Fig. S31), confirming conserved catalytic function.

### Regulatory mechanism of *AodTPSs*

Given the high expression of *AodTPS26* in *A. odorata*, the promoter regions (approximately 3000 bp) of *TPSs* from the three Meliaceae species were analyzed. The promoter of *AodTPS26* contained a greater number of ethylene response element (ERE) motifs compared to its orthologs in *A. indica* and *T. ciliata* (Fig. S32). These EREs are known binding sites for ethylene response factors (ERF) and activate *TPSs* transcription (Li et al. 2017, Wei et al. 2023, Zhao et al. 2023, Li et al. 2015). Further analysis revealed a total of 262 *ERFs* in *A. odorata*, substantially more than in *A. indica* (99) and *T. ciliata* (187) (Fig. S33), suggesting that ERF expansion in *A. odorata* potentially contributes to the enhanced *AodTPS* transcription and terpene biosynthesis.

Given the known role of ERF B3 subfamily members in activating *TPS* promoters (Li et al. 2015), highly expressed *A. odorata* ERFs from this subfamily were selected for further investigation (Fig. S34). Dual-luciferase assays in *N. benthamiana* showed that co-expression of the *AodTPS26* promoter with AodERF27 led to a strong fluorescent signal compared to the control (Fig. 6B), confirming that AodERF27 activates *AodTPS26* ttranscription. Yeast one-hybrid assay further supports this interaction: while both control strains Y187 (pAodTPS26-His2+AD) and Y187 (pAodTPS26-His2+AD-AodERF27) grew on SD/-Leu/-Trp medium, only the strain Y187 (pAodTPS26-His2+AD-AodERF27) grew on selective SD/-Leu/-Trp/-His medium (Fig. 6C). These results confirm that AodERF27 directly activates *AodTPS26*, contributing to the elevate expression of *AodTPSs* in *A. odorata*.

## Discussion

Phenotypic diversity in plants, including the abundance of secondary metabolites, arises from long-term genomic variations such as gene sequence mutations, genome duplications, chromosomal rearrangements, and gene loss (Clark et al. 2023, Soltis and Soltis 2020). In Meliaceae, these genomic events have profoundly affected metabolic profiles and medicinal traits, driving functional divergence among species. Analysis of essential oil from three representative Meliaceae species, *A. indica*, *T. ciliata*, and *A. odorata*, revealed marked differences in the composition and relative abundance of volatile compounds. Notably, *A. odorata* exhibited exceptionally high diversity and concentration of terpenes, likely contributing to its distinctive aroma and pharmacological properties. These findings suggest that interspecific genomic variation within Meliaceae plays a significant role in shaping chemical diversity. Despite *A. odorata*’s notable secondary metabolite profile, the lack of genomic resources has limited insight into the genetic basis of these traits. To address this, we generated a high-quality reference genome for *A. odorata* using a combination of Illumina, Nanopore, HiFi, and Hi-C sequencing technologies. The resulting assemblies were 462.31 Mb with contig N50 size of 12.10 Mb for Hap1, and 448.91 Mb with contig N50 size of 10.42 Mb for Hap2. This genome provides a valuable foundation for comparative genomics and the characterization of functional genes with in the Meliaceae family.

Polyploidy has played a pivotal role in shaping the genomic architecture and species diversity within the Meliaceae family. A recent WGD event was shared by *T*. *ciliata*, *T. sinensis*, and *X. granatum* (He et al. 2022, Wang et al. 2022, Ji et al. 2021), following an ancient WGT event occurred in core eudicots. Our study further identified a lineage-specific WGT event in *A. odorata*, which occurred approximately 28.49 Mya. WGD events introduced additional genome sets, forming subgenomes and leading to large-scale gene duplications (Wu et al. 2020). For instance, a lineage-specific WGT event in cabbage, nearly doubled its protein-coding gene count compared to *Arabidopsis thaliana*, which lacks recent WGD/WGT events (Hou et al. 2022). In contrast, genomic analyses of five Meliaceae species revealed that most retain approximately 33,000 genes, suggesting widespread gene loss after polyploidy. This is consistent with previous studies reporting that chromosomal rearrangements and pseudogenization during diploidization accelerate gene loss following polyploidization (Yin et al. 2023, Liu et al. 2014). In our study, extensive chromosomal rearrangements were observed among species and subgenomes after speciation and polyploid events, likely contributing to this loss. Additionally, recent LTR bursts in WGD/WGT lineage (*T. ciliata* and *A. odorata*) imply that post-polyploid genomic remodeling may mitigate the deleterious effects of TE insertion, promote genome plasticity that enhances LTR activity (Bourque et al. 2018).

Polyploidization plays a pivotal role in plant evolution by providing abundant genetic variations, thereby contributing to genomic complexity and functional diversification (Zhang et al. 2019). The subsequent diploidization process following WGD/WGT events further shapes phenotypic traits and environmental adaptability (Dodsworth et al. 2016). In our study, comprehensive analysis of gene retention, LTR insertion, and gene expression across Meliaceae subgenomes revealed subgenome dominance in *A. odorata*. Specifically, the LF subgenome retained more genes and exhibited lower frequencies of LTR insertions compared to MF1 and MF2, which may underlie its elevated gene expression and adaptive significance. Gene loss or pseudogenization following WGD or WGT likely contributes to genome stabilization and compaction. In this study, we found that gene families related to “sesquiterpene and triterpene biosynthesis pathways” were contracted in *A. odorata*, whereas adaptation-related genes were preferentially retained after WGD/WGT events, suggesting a selective retention strategy that supports environmental adaptability. These lineage-specific polyploidization events and their downstream genomic consequences may have been key drivers of phenotypic and metabolic divergence across Meliaceae species.

*A. odorata* stands out among Meliaceae for its high terpene content. Despite undergoing a lineage-specific WGT, several key genes involved in the MVA and MEP pathways, such as PKM and MDS, remained only 1–2 copies, implying strong functional constraint and selective loss of redundant copies (Jiao et al. 2011). Notably, contrary to the expected gene expansion after WGD/WGT, the *TPS* gene family was contracted in *A. odorata*. Previous studies suggest that functionally redundant gene copies are often lost during diploidization, streamlining the genome and reducing the burden of deleterious mutations (Innan and Kondrashov 2010). This process may also favor the enhanced expression of retained, functional alleles. While *TPSs* expanded in *A. indica* and *T. ciliata*, most exhibited low or negligible expression, possible due to dosage-balance constraints. In contrast, *A. odorata*, despite having fewer genes, showed consistently high expression levels of remained *AodTPSs*, suggesting transcriptional upregulation as a compensatory mechanism. Functionally, *AodTPS25*, the most highly expressed *AodTPSs* in leaves, catalyzes the production of caryophyllene from FPP, aligning with the dominant volatile in *A. odorata* leaves essential oil. *AodTPS21*, a species-specific gene highly expressed in stems, synthesize α-selinene. Although α-selinene was not directly detected, its likely derivative, Selina-3,7(11)-diene, was detected, implying that *AodTPS21* contributes indirectly to the characteristic odor profile. In addition, *AodTPS24*, a TPS-b subfamily member expressed in both leaves and stems, produces β-pinene. While its relatively low expression may explain sub-threshold product accumulation, its enzymatic role provides further insight into the diversity of monoterpenes in *A. odorata*. Collectively, the high expression of key *AodTPS* genes despite overall family contraction illustrates an adaptive strategy wherein gene dosage is balanced by enhanced transcription. This highlights a novel evolutionary trajectory in *A. odorata* following polyploidization, where functional optimization rather than gene expansion supports specialized metabolite production.

Notably, AodTPS26 and its ortholog Indica_017010 in *A. indica* and Tci19G002100 in *T. ciliata—*are all capable of catalyzing α-farnesene biosynthesis, a precursor of farnesol. However, only *AodTPS26* exhibits a uniquely high tissue-specific expression pattern, correlating with the exclusive accumulation of farnesol in *A. odorata*, and contributing to its unique secondary metabolite profile. Promoter specificity plays a central role in regulating gene expression by modulating transcription factor binding (He et al. 2023). The promoter of *AodTPS26* in *A. odorata* is notable enriched with ERE motifs, which act as binding sites for ERF transcription factors, a key family involved in plant development and environmental response (Wu et al. 2022). Genomic analysis revealed a substantial expansion of the ERF gene family in *A. odorata*, potentially underpinning the high expression of *AodTPS26*. Among these, AodERF27 was experimentally confirmed to directly bind to the *AodTPS26* promoter through Dual-luciferase assays and Yeast one-hybrid systems, providing strong evidence of its role in transcriptional activation of α-farnesene biosynthesis in *A. odorata*. Based on these findings, we propose the following regulatory model: A lineage-specific WGT event in *A. odorata* led to the expansion of the ERF members. Certain ERF members (e.g., AodERF27) acquired novel regulatory roles, such as activating AodTPS26, thereby remodeling the transcriptional regulation of the terpene biosynthesis pathways in *A. odorata*.

In conclusion, by generating a high-quality genome assembly of *A. odorata* and performing comparative genomic analyses with *A. indica* and *T. ciliata*, we identified a lineage-specific WGT and uncovered evidence of subgenome dominance in *A. odorata.* Notably, despite the contraction of the *TPS*s following WGT, transcriptional upregulation of retained *AodTPSs* effectively compensated for copy number reduction, resulting in enhanced terpenoid diversity. These findings highlight a unique evolutionary strategy in *A. odorata*, whereby regulatory innovation offsets gene loss to maintain specialized metabolic functions.

## Materials and methods

### Volatile terpenoid analysis

Volatile terpenoids were extracted from the fresh leaves and stems of *A. odorata*, *A. indica* and *T. ciliata* through hydro-distillation as previously described (Wu et al. 2014). Briefly, 500 g leaves or stems were soaked in distilled water and boiled for 4 h in a round-bottom flask fitted with a condenser. N-hexane containing n-pentadecane as an internal standard was used for extracting the obtained distillate. After filtration, the samples were analyzed using an Agilent 7890a gas chromatograph/5975c mass selective detector with a DB-5ms column. A total of 1.0 μl was injected without splitting. Helium was used as the carrier gas at a 1.2 ml/min flow rate. The oven temperature conditions were as follows: the initial temperature was 60 °C, which was increased to 135 °C at a rate of 10°C/min, then to 160 °C at a rate of 2°C/min, and finally to 260 °C at a rate of 10°C/min, where it remained for 5 min. The electron impact (EI) mode was used for the MS analysis. The ionizing energy was set to 70 eV, and full-scan mass spectra were collected within the scan range of 50 to 550 amu, followed by identification of compounds on the basis of NIST98 standard spectral libraries.

### Plant materials and genome sequencing

The sequenced *A. odorata* individual was collected from Xishuangbanna Tropical Botanical Garden, Chinese Academy of Sciences, Yunnan Province, China. Genomic DNA was extracted from young leaves of *A. odorata* using the QIAGEN Genomic Kit. DNA purity was assessed using a NanoDrop One UV-Vis spectrophotometer (Thermo Fisher Scientific, USA). A PacBio Sequel II instrument was used for HiFi sequencing. For Illumina paired-end sequencing, DNA libraries with a 350-bp insert size were constructed and then sequenced on the Illumina Hiseq Xte platform to produce short-read sequencing data. Then, 20-kb Nanopore libraries were constructed and sequenced through single molecule DNA sequencing on a Nanopore PromethION sequencer instrument. The Hi-C libraries were constructed from 150 paired-end reads with Dpnll cutter restriction enzyme and subsequently sequenced on Illumina NovaSeq. Raw Illumina sequencing reads were filtered with the default parameters of Fastp v0.20.0 (Chen et al. 2018) to obtain clean data for the genome assembly.

### *De novo* assembly and assessment

The genome assembly involved assembling, polishing and Hi-C scaffolding. First, the raw Nanopore reads were error-corrected in Canu v2.1.1 (Koren et al. 2017) with corrected Error Rate set to 0.045 to get consistent sequences. The Nanopore-corrected data were assembled into contigs with Nextdenovo v2.4.0 by using “-m 10 g -t 8 -p 8 -a 1” (https://github.com/Nextomics/NextDenovo). Hifiasm v0.19.5 (Cheng et al. 2022) was used to assemble the primary genome using HiFi, Nanopore and Hi-C sequencing data. Then clean Illumina data were further polished by alignment to the primary assembly for three rounds by using Nextpolish (Hu et al. 2020) with the parameters “-max_depth 100 -bwa -min_read_len 1k -max_depth 60 -x map-ont”. For Hi-C scaffolding, the clean Hi-C reads were mapped onto the contig assembly by using Bwa-mem2 v2.2 (Li, 2013). Using 3D-DNA v201008 (Dudchenko et al. 2017) to cluster, reorder and orient contigs onto pseudochromosomes. Manual adjustments were conducted with juicebox v1.11.08 (Durand et al. 2016) to obtain the chromosome-level genome of *A. odorata*. The removal of redundancy by using purge_dups v1.2.5 (Guan et al. 2020). The gaps were filled by quartet (Lin et al. 2023) using the assembly generated by Nextdenovo. Completeness was evaluated using Benchmarking Universal Single Copy Orthologs (BUSCO) (Simão et al. 2015)) with default parameters.

### Transcriptome sequencing and analysis

After three biological replicates each of *A. odorata, A. indica* and *T. ciliata* leaves and stems and *A. odorata* flowers were collected, RNA was extracted from samples by using RNAiso Plus (Qiagen). Sequencing was conducted on the Illumina HiSeq 2500 platform. The raw data were filtered using Fastp v0.20.0 (Chen et al. 2018) to obtain the clean data. The data were then mapped to the genome by using STAR v2.7.10a (Dobin et al. 2012), and the Transcripts Per Kilobase of exon model per Million mapped reads (TPM) values were calculated using RSEM v1.3.1 (Dewey and Bo 2011) with default parameters.

### Genome annotation

Repeat element was identified using EDTA (Ou et al. 2019), and the identified TEs were classified using TEsorter (Zhang et al. 2022) and DeepTE (Yan et al. 2020). TEs in the genome were then soft-masked using RepeatMasker (Price et al. 2005). Based on the soft-masked genome, genes were predicted by integrating homology-based, transcriptome-based and *de novo* prediction approaches by BRAKER v2.1.6 (Bruna et al. 2021) pipeline. Briefly, protein sequences from a homologous protein library (OrthoDB v10 (Kriventseva et al. 2019)) were aligned to the assembly by ProtHint v2.6.0. Transcriptome data from the leaves, stems and flowers were mapped to the genome by using HISAT2 v2.2.1 (Kim et al. 2015). Based on homologous proteins and the transcriptome, model training was conducted by AUGUSTUS v3.4.0 (Stanke et al. 2008) with default parameters. The *de novo* prediction was performed in GeneMark-ESSuiteversion4.69_lic (Bruna et al. 2020). Gene models were integrated with TSEBRA v1.0.3 (Gabriel et al. 2021), and corrected and formatted annotation files were generated using MAKER v3.1.4 (Campbell et al. 2014) and EVidenceModeler, respectively.

Non-coding RNAs (ncRNAs) were annotated using Cmscan by searching against the Rfam database (v9.1) (Griffiths-Jones et al. 2005). Gene functions were annotated according to eggnog-mapper (Cantalapiedra et al. 2021) and proteins were aligned against the KEGG, GO, SwissProt and NR database with Diamond v2.0.11.149 (Buchfink et al. 2021) (E-value <1e-10). Furthermore, the integrity of the genome assembly and annotation was assessed using BUSCO (Simão et al. 2015).

### Genome evolution

Gene family clusters were identified using OrthoFinder v2.3.12 (Emms and Kelly 2015) on the basis of multiple sequence alignment among 10 plant species, including *Acer pseudosieboldianum* (Li et al. 2022), *A. odorata*, *A. indica* (Du et al. 2022), *Carica papaya* (Yue et al. 2022), *Citrus sinensis* (Wu et al. 2023), *Litchi chinensis* (Hu et al. 2022), *T. ciliata* (Wang et al. 2022), *T. sinensis* (Ji et al. 2021), *X. granatum* (He et al. 2022) and *Zanthoxylum armatum* (Wang et al. 2021). Proteins encoded by single-copy gene families were extracted and aligned by Mafft v7.313 (Rozewicki et al. 2019), and then concatenated to a super alignment matrix. A maximum likelihood (ML) phylogenetic tree was constructed according to the GTRGAMMA model of RAxML with 200 bootstrap replicates. MCMCtree in PAML v4.10 (Yang 2007) was employed to determine the divergence time for the 10 species on the basis of correlated rates and the HKY85 model. Additionally, multiple fossil times from the TimeTree database (Kumar et al. 2017) were used as constraints for calibrating the time of the tree.

Based on OrthoFinder results, gene family expansions and contractions were identified using CAFE5 (Mendes et al. 2020). By referring to the protein alignments results, WGDI (Sun et al. 2022) was used to identify homologous gene pairs and calculate the synonymous substitutions site (Ks) (“-kp”) and syntenic blocks (“-icl”). Furthermore, WGD and speciation events were inferred from the *Ks* peaks of paralogous and orthologous genes, respectively. The neutral substitution rate (*r*) was calculated using the divergence time (*T*) and *Ks* peaks of *A. odorata* vs. *A. indica* as follows: *r* =*Ks*/*2T*.

### Construction of triplicated subgenomic blocks

Triplicated subgenomes were constructed using WGDI (Sun et al. 2022) basing on the ancestral core eudicots karyotypes (ACEK) identified by Sun *et al* (https://github.com/SunPengChuan/Angiosperm-karyotype-evolution). As previously described (Ma et al. 2021), according to chromosome complementarity and gene retention, the ACEKs were mapped to the *A. odorata* genome (“-km”) to divide it into different subgenomes. Then, syntenic genes were obtained by WGDI with the parameters “-pc -a”. Finally, a phylogenetic tree was constructed to examine the results (“-at”). In addition, based on the obtained syntenic genes, JCVI (Li 2013) (parameters: -minsize=3 -minspan=10) was used to analyze genome-wide interspecies collinearity among *A. odorata*, *A. indica* and *T. ciliata*.

### Identification and evolutionary analysis of pseudogenes

Pseudogenes of *A. odorata* and four other Meliaceae species were identified as previously described (Xie et al. 2019, Yin et al. 2023). First, intergenic regions were generated by masking genic regions and repeat elements by using RepeatMasker (Price et al. 2005). Second, the intergenic regions having sequences similar to known proteins were identified by Exonerate (Slater and Birney 2005) (https://github.com/nathanweeks/exonerate). Third, pseudogenes were linked into contigs based on the distance between hits on the chromosome and the query protein, which was set to 50 bp. Fourth, realignment of contigs was conducted to accurately identify the sequence and positions of disablements using tfasty (Pearson et al. 1997) (“-A -m 3 ‘q’”). Finally, Exonerate (Slater and Birney 2005) was reused to identify pseudogene-functional paralog pairs.

### Genome duplication

Genome-wide duplications in Meliaceae species were identified to explore genome evolution. The varying modes of different duplicated gene pairs were identified using DupGen_finder (Qiao et al. 2019) with default parameters (https://github.com/qiao-xin/DupGen_finder), including WGD, transposed duplications (TRDs), tandem duplications (TDs), dispersed duplications (DSDs) and proximal duplications (PDs).

### Analysis of genes involved in terpene biosynthesis

Terpene biosynthesis genes of *Arabidopsis thaliana* (Vranova et al. 2013) were used as bait sequences, and local BLAST search against the Meliaceae genome (E-value < 10 ^−5^) was conducted to identify genes involved in terpene synthesis. The results of phylogenetic analysis (https://mafft.cbrc.jp/alignment/server/index.html) and protein BLAST (https://blast.ncbi.nlm.nih.gov/Blast.cgi) were combined to distinguish homologs on the basis of local blast reports.

To identify *TPS*s and the cytochrome P450 gene family in the Meliaceae genome, a simple HMM Search with Pfam domains (TPS: PF01397 and PF03936, P450: PF00067) was conducted using TBtools (Chen et al. 2020) software. Target hits with E-values < 10^−5^ were identified as candidate genes.

The TPS phylogenetic tree was constructed using MEGA7 (Kumar et al. 2016) with the maximum likelihood method and 1000 bootstrap replicates. Visualization of collinear genes was performed by Find Gene Block Evolutionary Path By Gene Pairs within TBtools (Chen et al. 2020) based on syntenic analysis results in WGDI (Sun et al. 2022) (“-icl”).

### Determination of TPS enzyme activities

To characterize in vitro TPS enzyme activity, codon-optimized full-length *TPS* gene sequences were constructed into the pET28a vector with His tags. Constructs were transformed into *E. coli* BL21 (DE3) with empty pET28a vector as a control. At OD_600_ of 0.6, recombinant proteins expression was induced with 0.8 mM IPTG at 16℃ overnight. Proteins were purified using the HyPur T Ni-NTA 6FF (His-Tag) PrePacked Gravity Column Kit. Enzyme activity assays were conducted in a 420 μl buffer (50 mM Tris-HCl, 10 mM MgCl_2_, 100 mM KCl and 5% glycerol), 5 mM DTT, 10 ng purified proteins and 10 μg GPP, GGPP, or FPP. The reaction solution was incubated for 2 h at 30 ℃, and analyzed by GC-MS.

### Dual-luciferase assays

The *AodTPS26* promoter sequence (approximately 3000 bp) were extracted and submitted to the website PlantCare (http://bioinformatics.psb.ugent.be/webtools/plantcare/html/) to predict cis-acting elements, and the result was visualized using TBtools (Chen et al. 2020). The *AodTPS26* promoter fragments were cloned into the *PRI101-LUC* vector to construct the reporter, respectively. Additionally, AodERF27 cDNA were inserted into the *PRI101-6FLAG* vector to generate effectors, respectively. The recombinant vectors were transformed into the *Agrobacterium* strain EHA105, respectively. Positive transformants were incubated for two days in LB solid medium containing 50 μg/ml kanamycin and 25 μg/ml rifampicin. The culture was centrifuged, resuspended and adjusted to OD_600_=0.6 using MMA buffer (10 mM 2-(N-morpholino) ethanesulfonic acid (MES), 10 mM MgCl_2_, 100 μM acetosyringone, pH 5.6). The mixed *Agrobacterium* strains were infiltrated into the tobacco leaves. Fluorescence was detected using an automatic luminescent image system.

### Yeast one-hybrid assay

The CDS of *AodERF27* was cloned into the vector pGADT7, the promoter of *AodTPS26* was iinserted to the vector *pHIS2*. The recombinant pAodTPS26-His2 and AD-AodERF27 plasmids were co-transformed into the Y187 yeast strain using the Y187-pHis2 Yeast One-Hybrid interaction proving kit (YH1011-10 T; Coolaber, Beijing, China).

## Supplementary Information

Supplementary Material 1: Figure S1 Venn diagram of essential oil categories in *A. indica, T. ciliata* and *A. odorata* leaves. Figure S2 PCA of essential oil categories in *A. indica, T. ciliata*, and *A. odorata* leaves. Figure S3 Heatmap of essential oil categories and content in *A. indica, A. odorata*, and *T. ciliata* leaves. Figure S4 Sample collection from *A. odorata* leaf, flower, and stem. Figure S5 Estimation of *A. odorata* genome size and heterozygosity using k-mer size of 21. Figure S6 The Hi-C heatmap of *A. odorata*. Figure S7 Gene family counts across *A. odorata* and 8 other Sapindales species and the outgroup species *C. papya*. Figure S8 Dot plots syntenic blocks showing 1:3 chromosomal relationship between *C. sinensis* and *A. odorata* genome. Figure S9 Dot plots syntenic blocks showing 2:3 chromosomal relationship between *T. cilita* and *A. odorata* genome. Figure S10 Histogram of LTR insertion times in *A. indica, A. odorata*, and *T. ciliata*. Figure S11 Genes and pseudogenes counts in five Meliaceae species. Figure S12 KEGG enrichment of expanded gene families in *A. odorata*. Figure S13 KEGG enrichment of expanded gene families in *T. ciliata*. Figure S14 KEGG enrichment of expanded and contracted gene families in *A. indica*. Figure S15 Categories and numbers of duplicated genes in five Meliaceae species. Figure S16 Categories and numbers of duplicated genes in *A. odorata*. Figure S17 Segmental collinearity among *A. odorata*, *T. ciliata*, and *A. indica* based on ACEK blocks (A1-A7, B1-B7, C1-C7). Figure S18 Categories and numbers of LTR and DNA elements in the three *A. odorata* subgenomes. Figure S19 DNA density within 10 kb upstream and downstream of genes in *A. odorata* and *T. ciliata* subgenomes. Figure S20 LTR density within 10 kb upstream and downstream of genes in *T. ciliata* subgenomes. Figure S21 Histogram of LTR insertion times in the LF, MF1, and MF2 subgenomes of *A. odorata*. Figure S22 Box plots of homologous gene expression in leaves and stems of *T. ciliata* subgenomes. Figure S23 KEGG pathway enrichment analysis of three subgenomes gene families in *A. odorata*. Figure S24 Synteny of key genes in the MVA pathway for terpenoid biosynthesis in *A. indica, T. ciliata*, and *A. odorata*. Figure S25 Synteny of key genes in the MEP pathway for terpenoid biosynthesis in *A. indica, T. ciliata*, and *A. odorata*. Figure S26 Synteny of key genes for terpenoid precursor biosynthesis in *A. indica, T. ciliata*, and *A. odorata*. Figure S27 Synteny of key genes for terpenoid skeleton biosynthesis in *A. indica, T. ciliata*, and *A. odorata*. Figure S28 Heatmap of AodTPS24 and homologous gene expression. Figure S29 Heatmap of AodTPS26 and homologous gene expression. Figure S30 Predicted three-dimensional structures of AodTPS26, Indica_017010, and Tci19G002100. Figure S31 GC-MS spectra of volatile terpenes from overexpressed *AodTPS26, Tci19G002100*, and *Indica_017010*. Figure S32 Cis-element analysis of the 3000 bp promoter regions of *AodTPS26* and its homologs. Figure S33 The phylogenetic tree of ERF. Figure S34 Heatmap of ERF subfamily B3 gene expression in *A. odorata* leaves and stems.

Supplementary Material 2: Table S1 The genome sequencing data of *A. odorata*. Table S2 Summary statistics of genome assembly of *A. odorata*. Table S3 Pseudochromosome length statistics in *A. odorata* genome. Table S4 Genome BUSCO assessment for *A. odorata*. Table S5 Transposable elements content in Hap1 of the *A. odorata* genome. Table S6 Transposable elements content in Hap2 of the *A. odorata* genome. Table S7 Functional annotations of *A. odorata* genes. Table S8 Statistics of non-coding RNAs in the *A. odorata* genome. Table S9 Protein-level BUSCO assessment for *A. odorata*. Table S10 Comparison of published chromosome-level Meliaceae genomes. Table S11 Resequencing mapping rate for five individuals of *A. indica, T. ciliata* and *A. odorata*. Table S12 Gene retention ratios in subgenomes of *A. odorata* and *T. ciliata* based on ACEK blocks comparing with *A. indica.*

## Data Availability

All data supporting the results of this study are available within the manuscript and its Supplementary materials. The genome data generated from this study have been deposited in the National Genomics Data Center under accession number CRA020431. The genome assembly and annotation could be obtained from Figshare (10.6084/m9.figshare.27794091).

## Abbreviations

ACEK: Ancestral core eudicots karyotypes
DSD: Dispersed duplications
EI: Electron impact
ERE: Ethylene response element
ERF: Ethylene response factor
FPP: Farnesyl Pyrophosphate
GGPP: Geranylgeranyl Pyrophosphate
*Ka*: Nonsynonymous substitutions per site
*Ks*: Synonymous substitutions site
LF: Least-fractionated
MEP: Methylerythritol phosphate
MF: Max-fractionated
MF1: Medium-fractionated
MF2: Most-fractionated
ML: Maximum likelihood
MVA: Mevalonic acid
Mya: Million years ago
PCA: Principal component analysis
PD: Proximal duplications
r: Neutral substitution rate
T: Divergence time
T2T: telomere-to-telomere
TD: Tandem duplications
TE: Transposable element
TPM: Transcripts Per Kilobase of exon model per Million mapped reads
TPS: Terpene synthase-encoding gene
TRD: Transposed duplications
WGD: Whole-genome duplication
WGT: Whole-genome triplication

## References

[CR1] Bourque G, Burns KH, Gehring M, Gorbunova V, Seluanov A, Hammell M, et al. Ten things you should know about transposable elements. Genome Biol. 2018;19:199. 30454069 10.1186/s13059-018-1577-z PMC6240941

[CR2] Bruna T, Hoff KJ, Lomsadze A, Stanke M, Borodovsky M. BRAKER2: automatic eukaryotic genome annotation with GeneMark-EP+ and AUGUSTUS supported by a protein database. NAR Genom Bioinform. 2021;3:lqaa108. 33575650 10.1093/nargab/lqaa108 PMC7787252

[CR3] Bruna T, Lomsadze A, Borodovsky M. GeneMark-EP+: eukaryotic gene prediction with self-training in the space of genes and proteins. NAR Genom Bioinform. 2020;2:lqaa026. 32440658 10.1093/nargab/lqaa026 PMC7222226

[CR4] Buchfink B, Reuter K, Drost HG. Sensitive protein alignments at tree-of-life scale using DIAMOND. Nat Methods. 2021;18:366–8. 33828273 10.1038/s41592-021-01101-x PMC8026399

[CR5] Campbell MS, Holt C, Moore B, Yandell M. Genome annotation and curation using MAKER and MAKER-P. Curr Protoc Bioinformatics. 2014;48:4.11.1-39. 25501943 10.1002/0471250953.bi0411s48 PMC4286374

[CR6] Cantalapiedra CP, Hernandez-Plaza A, Letunic I, Bork P, Huerta-Cepas J. eggNOG-mapper v2: functional annotation, orthology assignments, and domain prediction at the metagenomic scale. Mol Biol Evol. 2021;38:5825–9. 34597405 10.1093/molbev/msab293 PMC8662613

[CR7] Chen C, Chen H, Zhang Y, Thomas HR, Frank MH, He Y, et al. TBtools: an integrative toolkit developed for interactive analyses of big biological data. Mol Plant. 2020;13:1194–202. 32585190 10.1016/j.molp.2020.06.009

[CR8] Chen S, Zhou Y, Chen Y, Gu J. Fastp: an ultra-fast all-in-one FASTQ preprocessor. Bioinformatics. 2018;34:i884–90. 30423086 10.1093/bioinformatics/bty560 PMC6129281

[CR9] Cheng F, Wu J, Cai X, Liang J, Freeling M, Wang X. Gene retention, fractionation and subgenome differences in polyploid plants. Nat Plants. 2018;4:258–68. 29725103 10.1038/s41477-018-0136-7

[CR10] Cheng F, Wu J, Wang X. Genome triplication drove the diversification of *Brassica* plants. Hortic Res. 2014;1:190–7. 10.1038/hortres.2014.24 PMC4596316 26504539

[CR11] Cheng H, Jarvis ED, Fedrigo O, Koepfli KP, Urban L, Gemmell NJ, et al. Haplotype-resolved assembly of diploid genomes without parental data. Nat Biotechnol. 2022;40:1332. 35332338 10.1038/s41587-022-01261-x PMC9464699

[CR12] Clark JW, Hetherington AJ, Morris JL, Pressel S, Duckett JG, Puttick MN, et al. Evolution of phenotypic disparity in the plant kingdom. Nat Plants. 2023;9:1618–26. 37666963 10.1038/s41477-023-01513-x PMC10581900

[CR13] Deb SK, Edger PP, Pires JC, McKain MR. Patterns, mechanisms, and consequences of homoeologous exchange in allopolyploid angiosperms: a genomic and epigenomic perspective. New Phytol. 2023;238:2284–304. 37010081 10.1111/nph.18927

[CR14] Dewey CN, Bo L. RSEM: accurate transcript quantification from RNA-Seq data with or without a reference genome. BMC Bioinf. 2011;12:323. 10.1186/1471-2105-12-323 PMC3163565 21816040

[CR15] Dobin A, Davis CA, Schlesinger F, Drenkow J, Gingeras TR. STAR: ultrafast universal RNA-seq aligner. Bioinformatics. 2012;29:15–21. 23104886 10.1093/bioinformatics/bts635 PMC3530905

[CR16] Dodsworth S, Chase MW, Leitch AR. Is post-polyploidization diploidization the key to the evolutionary success of angiosperms? Bot J Linn Soc. 2016;180:1–5.

[CR17] Du Y, Song W, Yin Z, Wu S, Liu J, Wang N, et al. Genomic analysis based on chromosome-level genome assembly reveals an expansion of terpene biosynthesis of *Azadirachta indica*. Front Plant Sci. 2022;13:853861. 35528946 10.3389/fpls.2022.853861 PMC9069239

[CR18] Duan D, Chen L, Yang X, Tu Y, Jiao S. Antidepressant-like effect of essential oil isolated from *Toona ciliata* Roem. var. *yunnanensis*. J Nat Med. 2015;69:191–7. 25465853 10.1007/s11418-014-0878-0

[CR19] Dudchenko O, Batra SS, Omer AD, et al. De novo assembly of the *Aedes aegypti* genome using Hi-C yields chromosome-length scaffolds. Science. 2017;356:92–5. 28336562 10.1126/science.aal3327 PMC5635820

[CR20] Durand NC, Robinson JT, Shamim MS, Machol I, Mesirov JP, Lander ES, et al. Juicebox provides a visualization system for Hi-C contact maps with unlimited zoom. Cell Syst. 2016;3:99–101. 27467250 10.1016/j.cels.2015.07.012 PMC5596920

[CR21] Edger PP, Heidel-Fischer HM, Bekaert M, Rota J, Glockner G, Platts AE, et al. The butterfly plant arms-race escalated by gene and genome duplications. Proc Natl Acad Sci U S A. 2015;112:8362–6. 26100883 10.1073/pnas.1503926112 PMC4500235

[CR22] Emms DM, Kelly S. OrthoFinder: solving fundamental biases in whole genome comparisons dramatically improves orthogroup inference accuracy. Genome Biol. 2015;16:157. 26243257 10.1186/s13059-015-0721-2 PMC4531804

[CR23] Gabriel L, Hoff KJ, Bruna T, Borodovsky M, Stanke M. TSEBRA: transcript selector for BRAKER. BMC Bioinf. 2021;22:566. 10.1186/s12859-021-04482-0 PMC8620231 34823473

[CR24] Greger H. Comparative phytochemistry of flavaglines (= rocaglamides), a group of highly bioactive flavolignans from *Aglaia* species (Meliaceae). Phytochem Rev. 2021;21:725–64. 34104125 10.1007/s11101-021-09761-5 PMC8176878

[CR25] Griffiths-Jones S, Moxon S, Marshall M, Khanna A, Bateman A. Rfam: annotating non-coding RNAs in complete genomes. Nucleic Acids Res. 2005;33:D121-4. 15608160 10.1093/nar/gki081 PMC540035

[CR26] Guan D, McCarthy SA, Wood J, Howe K, Wang Y, Durbin R. Identifying and removing haplotypic duplication in primary genome assemblies. Bioinformatics. 2020;36:2896–8. 31971576 10.1093/bioinformatics/btaa025 PMC7203741

[CR27] He H, Yang M, Li S, Zhang G, Ding Z, Zhang L, et al. Mechanisms and biotechnological applications of transcription factors. Synth Syst Biotechnol. 2023;8:565–77. 37691767 10.1016/j.synbio.2023.08.006 PMC10482752

[CR28] He Z, Feng X, Chen Q, Li L, Li S, Han K, et al. Evolution of coastal forests based on a full set of mangrove genomes. Nat Ecol Evol. 2022;6:738–49. 35484219 10.1038/s41559-022-01744-9

[CR29] Hofberger JA, Lyons E, Edger PP, Chris Pires J, Eric Schranz M. Whole genome and tandem duplicate retention facilitated glucosinolate pathway diversification in the mustard family. Genome Biol Evol. 2013;5:2155–73. 24171911 10.1093/gbe/evt162 PMC3845643

[CR30] Hou X, Wang D, Cheng Z, Wang Y, Jiao Y. A near-complete assembly of an *Arabidopsis thaliana* genome. Mol Plant. 2022;15:1247–50. 35655433 10.1016/j.molp.2022.05.014

[CR31] Hu G, Feng J, Xiang X, Wang J, Salojarvi J, Liu C, et al. Two divergent haplotypes from a highly heterozygous lychee genome suggest independent domestication events for early and late-maturing cultivars. Nat Genet. 2022;54:73–83. 34980919 10.1038/s41588-021-00971-3 PMC8755541

[CR32] Hu J, Fan J, Sun Z, Liu S. Nextpolish: a fast and efficient genome polishing tool for long-read assembly. Bioinformatics. 2020;36:2253–5. 31778144 10.1093/bioinformatics/btz891

[CR33] Innan H, Kondrashov F. The evolution of gene duplications: classifying and distinguishing between models. Nat Rev Genet. 2010;11:97–108. 20051986 10.1038/nrg2689

[CR34] Ji YT, Xiu Z, Chen CH, Wang Y, Yang JX, Sui JJ, et al. Long read sequencing of *Toona sinensis* (A. Juss) Roem: A chromosome-level reference genome for the family Meliaceae. Mol Ecol Resour. 2021;21:1243–55. 33421343 10.1111/1755-0998.13318

[CR35] Jiao Y, Wickett NJ, Ayyampalayam S, Chanderbali AS, Landherr L, Ralph PE, et al. Ancestral polyploidy in seed plants and angiosperms. Nature. 2011;473:97–100. 21478875 10.1038/nature09916

[CR36] Karunanithi PS, Zerbe P. Terpene synthases as metabolic gatekeepers in the evolution of plant terpenoid chemical diversity. Front Plant Sci. 2019;10:1166. 31632418 10.3389/fpls.2019.01166 PMC6779861

[CR37] Kato-Noguchi H, Suzuki M, Noguchi K, Ohno O, Suenaga K, Laosinwattana C. A Potent Phytotoxic Substance in *Aglaia odorata* Lour. Chem Biodivers. 2016;13:549–54. 27088639 10.1002/cbdv.201500175

[CR38] Khan HAA. Toxicity, repellent and oviposition deterrent effects of select essential oils against the house fly *Musca domestica*. J Asia-Pac Entomol. 2021;24:15–20.

[CR39] Kim D, Langmead B, Salzberg SL. HISAT: a fast spliced aligner with low memory requirements. Nat Methods. 2015;12:357–60. 25751142 10.1038/nmeth.3317 PMC4655817

[CR40] Koren S, Walenz BP, Berlin K, Miller JR, Bergman NH, Phillippy AM. Canu: scalable and accurate long-read assembly via adaptive k-mer weighting and repeat separation. Genome Res. 2017;27:722–36. 28298431 10.1101/gr.215087.116 PMC5411767

[CR41] Kriventseva EV, Kuznetsov D, Tegenfeldt F, Manni M, Dias R, Simao FA, et al. OrthoDB v10: sampling the diversity of animal, plant, fungal, protist, bacterial and viral genomes for evolutionary and functional annotations of orthologs. Nucleic Acids Res. 2019;47(D1):D807–11. 30395283 10.1093/nar/gky1053 PMC6323947

[CR42] Kumar S, Stecher G, Suleski M, Hedges SB. Timetree: a resource for timelines, timetrees, and divergence times. Mol Biol Evol. 2017;34:1812–9. 28387841 10.1093/molbev/msx116

[CR43] Kumar S, Stecher G, Tamura K. MEGA7: molecular evolutionary genetics analysis version 7.0 for bigger datasets. Mol Biol Evol. 2016;33:1870–4. 27004904 10.1093/molbev/msw054 PMC8210823

[CR44] Li H. Aligning sequence reads, clone sequences and assembly contigs with BWA-MEM. Genomics. 2013;27:314–24.

[CR45] Li S, Wang H, Li F, Chen Z, Li X, Zhu L, et al. The maize transcription factor EREB58 mediates the jasmonate-induced production of sesquiterpene volatiles. Plant J. 2015;84:296–308. 26303437 10.1111/tpj.12994

[CR46] Li X, Cai K, Han Z, Zhang S, Sun A, Xie Y, et al. Chromosome-level genome assembly for *Acer pseudosieboldianum* and highlights to mechanisms for leaf color and shape change. Front Plant Sci. 2022;13:850054. 35310631 10.3389/fpls.2022.850054 PMC8927880

[CR47] Li X, Xu Y, Shen S, Yin X, Klee H, Zhang B, et al. Transcription factor CitERF71 activates the terpene synthase gene *CitTPS16* involved in the synthesis of E-geraniol in sweet orange fruit. J Exp Bot. 2017;68:4929–38. 28992329 10.1093/jxb/erx316 PMC5853461

[CR48] Lin Y, Ye C, Li X, Chen Q, Wu Y, Zhang F, et al. quarTeT: a telomere-to-telomere toolkit for gap-free genome assembly and centromeric repeat identification. Hortic Res. 2023;10:2662–6810. 10.1093/hr/uhad127 PMC10407605 37560017

[CR49] Liu S, Liu Y, Yang X, Tong C, Edwards D, Parkin IA, et al. The *Brassica oleracea* genome reveals the asymmetrical evolution of polyploid genomes. Nat Commun. 2014;5:3930. 24852848 10.1038/ncomms4930 PMC4279128

[CR50] Ma J, Sun P, Wang D, Wang Z, Yang J, Li Y, et al. The *Chloranthus sessilifolius* genome provides insight into early diversification of angiosperms. Nat Commun. 2021;12:6929. 34836967 10.1038/s41467-021-26931-3 PMC8626421

[CR51] Mandakova T, Lysak MA. Post-polyploid diploidization and diversification through dysploid changes. Curr Opin Plant Biol. 2018;42:55–65. 29567623 10.1016/j.pbi.2018.03.001

[CR52] Mendes FK, Vanderpool D, Fulton B, Hahn MW. CAFE 5 models variation in evolutionary rates among gene families. Bioinformatics. 2020;36:22–3. 10.1093/bioinformatics/btaa1022 33325502

[CR53] Moriyama Y, Koshiba-Takeuchi K. Significance of whole-genome duplications on the emergence of evolutionary novelties. Brief Funct Genomics. 2018;17:329–38. 29579140 10.1093/bfgp/ely007

[CR54] Ou S, Su W, Liao Y, Chougule K, Agda JRA, Hellinga AJ, et al. Benchmarking transposable element annotation methods for creation of a streamlined, comprehensive pipeline. Genome Biol. 2019;20:275. 31843001 10.1186/s13059-019-1905-y PMC6913007

[CR55] Pearson WR, Wood T, Zhang Z, Miller W. Comparison of DNA sequences with protein sequences. Genomics. 1997;46:24–36. 9403055 10.1006/geno.1997.4995

[CR56] Price AL, Jones NC, Pevzner PA. De novo identification of repeat families in large genomes. Bioinformatics. 2005;21:1351–8. 10.1093/bioinformatics/bti1018 15961478

[CR57] Qiao X, Li Q, Yin H, Qi K, Li L, Wang R, et al. Gene duplication and evolution in recurring polyploidization-diploidization cycles in plants. Genome Biol. 2019;20:38. 30791939 10.1186/s13059-019-1650-2 PMC6383267

[CR58] Qiao X, Zhang S, Paterson AH. Pervasive genome duplications across the plant tree of life and their links to major evolutionary innovations and transitions. Comput Struct Biotechnol J. 2022;20:3248–56. 35782740 10.1016/j.csbj.2022.06.026 PMC9237934

[CR59] Rozewicki J, Li S, Amada KM, Standley DM, Katoh K. Mafft-dash: integrated protein sequence and structural alignment. Nucleic Acids Res. 2019;47:W5–10. 31062021 10.1093/nar/gkz342 PMC6602451

[CR60] Simão FA, Waterhouse RM, Panagiotis I, Kriventseva EV, Zdobnov EM. BUSCO: assessing genome assembly and annotation completeness with single-copy orthologs. Bioinformatics. 2015;31:3210–2. 26059717 10.1093/bioinformatics/btv351

[CR61] Slater GS, Birney E. Automated generation of heuristics for biological sequence comparison. BMC Bioinf. 2005;6:31. 10.1186/1471-2105-6-31 PMC553969 15713233

[CR62] Soltis PS, Soltis DE. Ancient WGD events as drivers of key innovations in angiosperms. Curr Opin Plant Biol. 2016;30:159–65. 27064530 10.1016/j.pbi.2016.03.015

[CR63] Soltis PS, Soltis DE. Plant genomes: markers of evolutionary history and drivers of evolutionary change. Plants People Planet. 2020;3:74–82.

[CR64] Stanke M, Diekhans M, Baertsch R, Haussler D, et al. Using native and syntenically mapped cDNA alignments to improve de novo gene finding. Bioinformatics. 2008;24:637–44. 18218656 10.1093/bioinformatics/btn013

[CR65] Sun P, Jiao B, Yang Y, Shan L, Li T, Li X, et al. WGDI: a user-friendly toolkit for evolutionary analyses of whole-genome duplications and ancestral karyotypes. Mol Plant. 2022;15:1841–51. 36307977 10.1016/j.molp.2022.10.018

[CR66] Tundis R, Loizzo MR, Menichini F. An overview on chemical aspects and potential health benefits of limonoids and their derivatives. Crit Rev Food Sci Nutr. 2014;54:225–50. 24188270 10.1080/10408398.2011.581400

[CR67] Vranova E, Coman D, Gruissem W. Network analysis of the MVA and MEP pathways for isoprenoid synthesis. Annu Rev Plant Biol. 2013;64:665–700. 23451776 10.1146/annurev-arplant-050312-120116

[CR68] Wang C-L, Shi J-X, Wu Y. Chemical and antimicrobial analyses of essential oil of *Toona sinensis* from China. Asian J Chem. 2014;26:2557–60.

[CR69] Wang M, Tong S, Ma T, Xi Z, Liu J. Chromosome-level genome assembly of Sichuan pepper provides insights into apomixis, drought tolerance, and alkaloid biosynthesis. Mol Ecol Resour. 2021;21:2533–45. 34145765 10.1111/1755-0998.13449

[CR70] Wang X, Xiao Y, He ZH, Li LL, Song HY, Zhang JJ, et al. A chromosome-level genome assembly of *Toona ciliata* (Meliaceae). Genome Biol Evol. 2022;14:evac121. 35880739 10.1093/gbe/evac121 PMC9348625

[CR71] Wei J, Yang Y, Peng Y, Wang S, Zhang J, Liu X, et al. Biosynthesis and the transcriptional regulation of terpenoids in tea plants (*Camellia sinensis*). Int J Mol Sci. 2023;24:6937. 37108101 10.3390/ijms24086937 PMC10138656

[CR72] Weyerstahl P, Marschall H, Son PT, Giang PM. Constituents of the flower essential oil of *Aglaia odorata* Lour. from Vietnam. Flavour Fragrance J. 1999;14:219–24.

[CR73] Wu B, Yu Q, Deng Z, Duan Y, Luo F, Gmitter F Jr. A chromosome-level phased genome enabling allele-level studies in sweet orange: a case study on citrus Huanglongbing tolerance. Hortic Res. 2023;10:uhac247. 36643761 10.1093/hr/uhac247 PMC9832951

[CR74] Wu JG, Peng W, Yi J, Wu YB, Chen TQ, Wong KH, et al. Chemical composition, antimicrobial activity against *Staphylococcus aureus* and a pro-apoptotic effect in SGC-7901 of the essential oil from *Toona sinensis* (A. Juss.) Roem. leaves. J Ethnopharmacol. 2014;154:198–205. 24726685 10.1016/j.jep.2014.04.002 PMC7126815

[CR75] Wu S, Han B, Jiao Y. Genetic contribution of paleopolyploidy to adaptive evolution in angiosperms. Mol Plant. 2020;13:59–71. 31678615 10.1016/j.molp.2019.10.012

[CR76] Wu Y, Li X, Zhang J, Zhao H, Tan S, Xu W, et al. ERF subfamily transcription factors and their function in plant responses to abiotic stresses. Front Plant Sci. 2022;13:1042084. 36531407 10.3389/fpls.2022.1042084 PMC9748296

[CR77] Xie J, Li Y, Liu X, Zhao Y, Li B, Ingvarsson PK, et al. Evolutionary origins of pseudogenes and their association with regulatory sequences in plants. Plant Cell. 2019;31:563–78. 30760562 10.1105/tpc.18.00601 PMC6482637

[CR78] Yan H, Bombarely A, Li S. DeepTE: a computational method for de novo classification of transposons with convolutional neural network. Bioinformatics. 2020;36:4269–75. 32415954 10.1093/bioinformatics/btaa519

[CR79] Yang Z. PAML 4: phylogenetic analysis by maximum likelihood. Mol Biol Evol. 2007;24(8):1586–91. 17483113 10.1093/molbev/msm088

[CR80] Yin X, Yang D, Zhao Y, Yang X, Zhou Z, Sun X, et al. Differences in pseudogene evolution contributed to the contrasting flavors of turnip and Chiifu, two *Brassica rapa* subspecies. Plant Commun. 2023;4:100427. 36056558 10.1016/j.xplc.2022.100427 PMC9860189

[CR81] Yue J, VanBuren R, Liu J, Fang J, Zhang X, Liao Z, et al. Sunup and sunset genomes revealed impact of particle bombardment mediated transformation and domestication history in papaya. Nat Genet. 2022;54:715–24. 35551309 10.1038/s41588-022-01068-1

[CR82] Zhang K, Wang X, Cheng F. Plant polyploidy: origin, evolution, and its influence on crop domestication. Hortic Plant J. 2019;5:231–9.

[CR83] Zhang RG, Li GY, Wang XL, Dainat J, Wang ZX, Ou S, et al. TEsorter: an accurate and fast method to classify LTR-retrotransposons in plant genomes. Hortic Res. 2022;9:uhac017. 35184178 10.1093/hr/uhac017 PMC9002660

[CR84] Zhao Y, Wang M, Chen Y, Gao M, Wu L, Wang Y. LcERF134 increases the production of monoterpenes by activating the terpene biosynthesis pathway in *Litsea cubeba*. Int J Biol Macromol. 2023;232:123378. 36716839 10.1016/j.ijbiomac.2023.123378

