# Supplementary material for: High-quality genome assembly of Aglaia odorata reveals evolution, terpenes diversity and abundance in Meliaceae
Source: Mol Hortic. 2026 Apr 9;6:29. doi: 10.1186/s43897-025-00212-9 (PMC13063475; doi:10.1186/s43897-025-00212-9)
Supplement: Supplementary file 1 — Supplementary Material 1: Figure S1 Venn diagram of essential oil categories in A. indica, T. ciliata and A. odorata leaves. Figure S2 PCA of essential oil categories in A. indica, T. ciliata, and A. odorata leaves. Figure S3 Heatmap of essential oil categories and content in A. indica, A. odorata, and T. ciliata leaves. Figure S4 Sample collection from A. odorata leaf, flower, and stem. Figure S5 Estimation of A. odorata genome size and heterozygosity using k-mer size of 21. Figure S6 The Hi-C heatmap of A. odorata. Figure S7 Gene family counts across A. odorata and 8 other Sapindales species and the outgroup species C. papya. Figure S8 Dot plots syntenic blocks showing 1:3 chromosomal relationship between C. sinensis and A. odorata genome. Figure S9 Dot plots syntenic blocks showing 2:3 chromosomal relationship between T. cilita and A. odorata genome. Figure S10 Histogram of LTR insertion times in A. indica, A. odorata, and T. ciliata. Figure S11 Genes and pseudogenes counts in five Meliaceae species. Figure S12 KEGG enrichment of expanded gene families in A. odorata. Figure S13 KEGG enrichment of expanded gene families in T. ciliata. Figure S14 KEGG enrichment of expanded and contracted gene families in A. indica. Figure S15 Categories and numbers of duplicated genes in five Meliaceae species. Figure S16 Categories and numbers of duplicated genes in A. odorata. Figure S17 Segmental collinearity among A. odorata, T. ciliata, and A. indica based on ACEK blocks (A1-A7, B1-B7, C1-C7). Figure S18 Categories and numbers of LTR and DNA elements in the three A. odorata subgenomes. Figure S19 DNA density within 10 kb upstream and downstream of genes in A. odorata and T. ciliata subgenomes. Figure S20 LTR density within 10 kb upstream and downstream of genes in T. ciliata subgenomes. Figure S21 Histogram of LTR insertion times in the LF, MF1, and MF2 subgenomes of A. odorata. Figure S22 Box plots of homologous gene expression in leaves and stems of T. ciliata subgenomes. F [file 43897_2025_212_MOESM1_ESM.pdf]

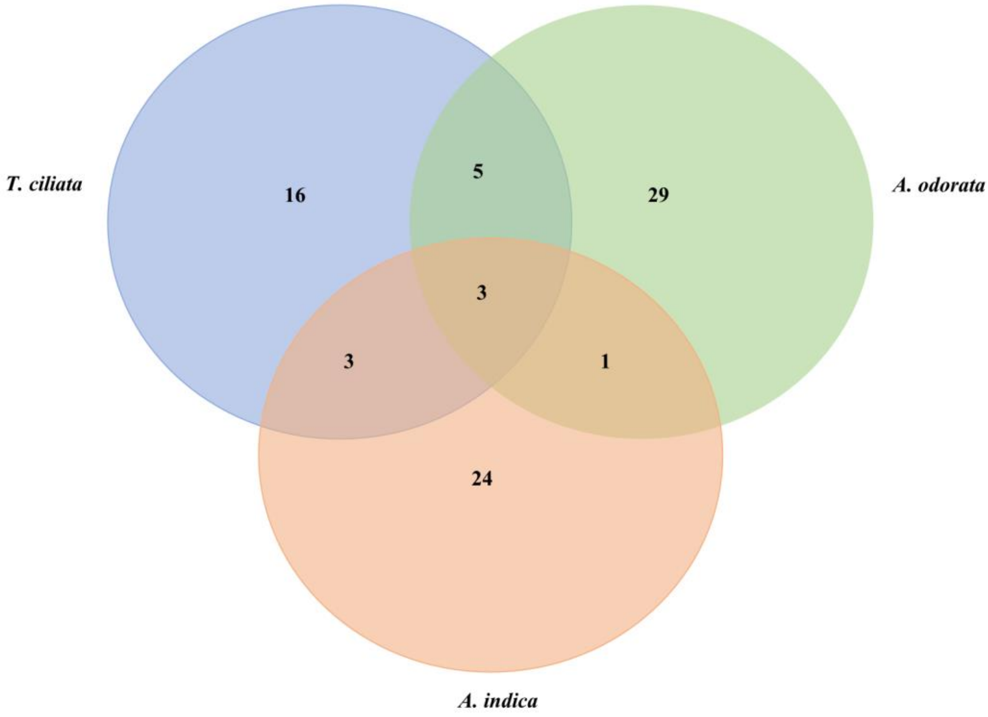

Figure S1 Venn diagram of essential oil categories in *A. indica*, *T. ciliata* and *A. odorata* leaves.

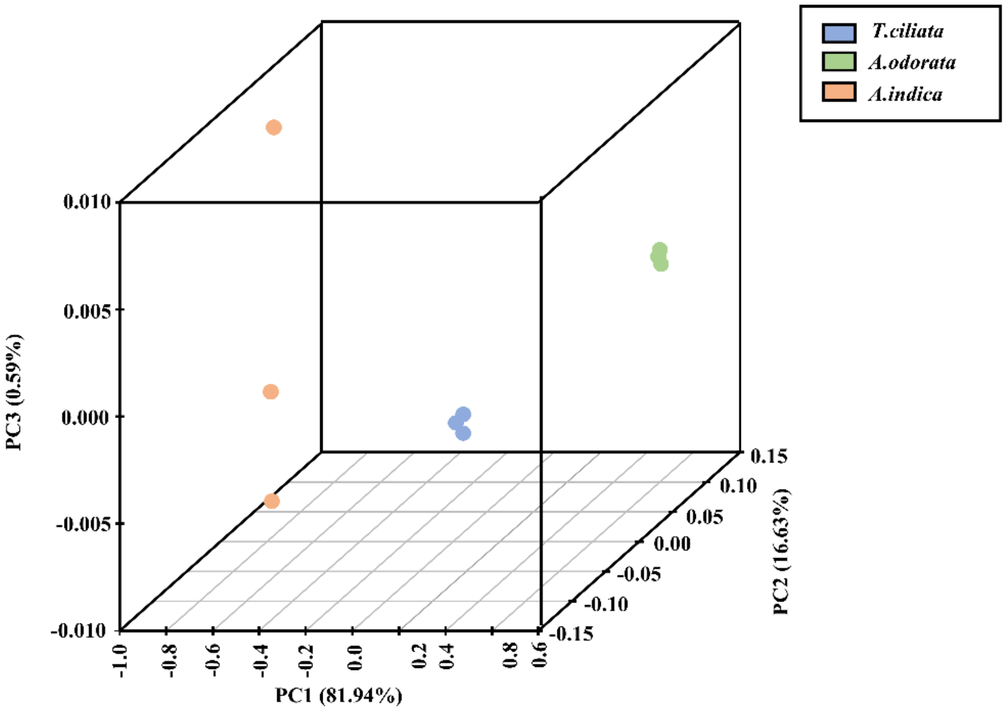

Figure S2 PCA of essential oil categories in *A. indica*, *T. ciliata*, and *A. odorata* leaves.

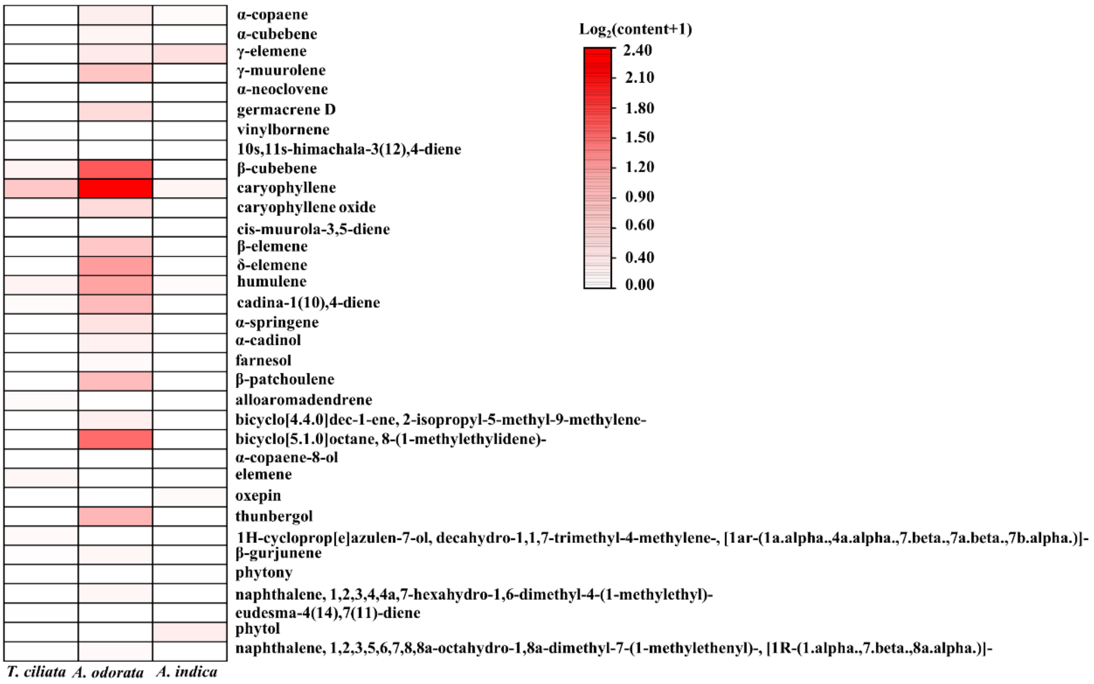

Figure S3 Heatmap of essential oil categories and content in *A. indica, A. odorata*, and *T. ciliata* leaves.

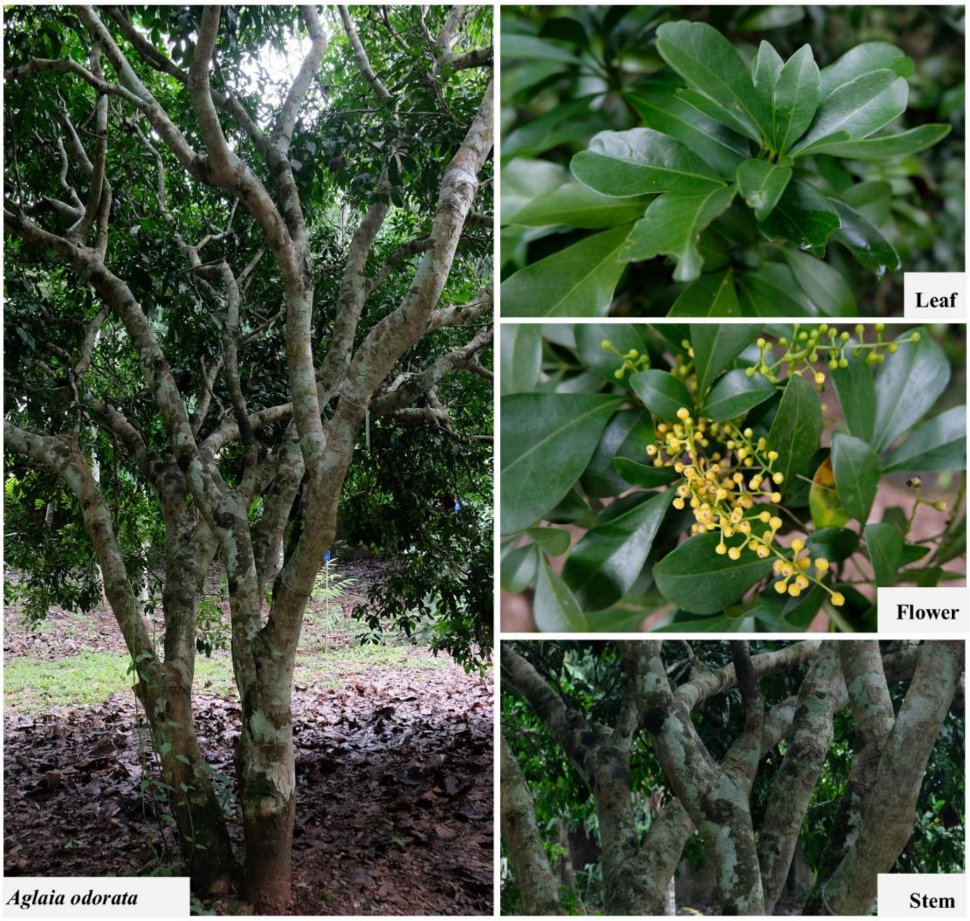

Figure S4 Sample collection from *A. odorata* leaf, flower, and stem.

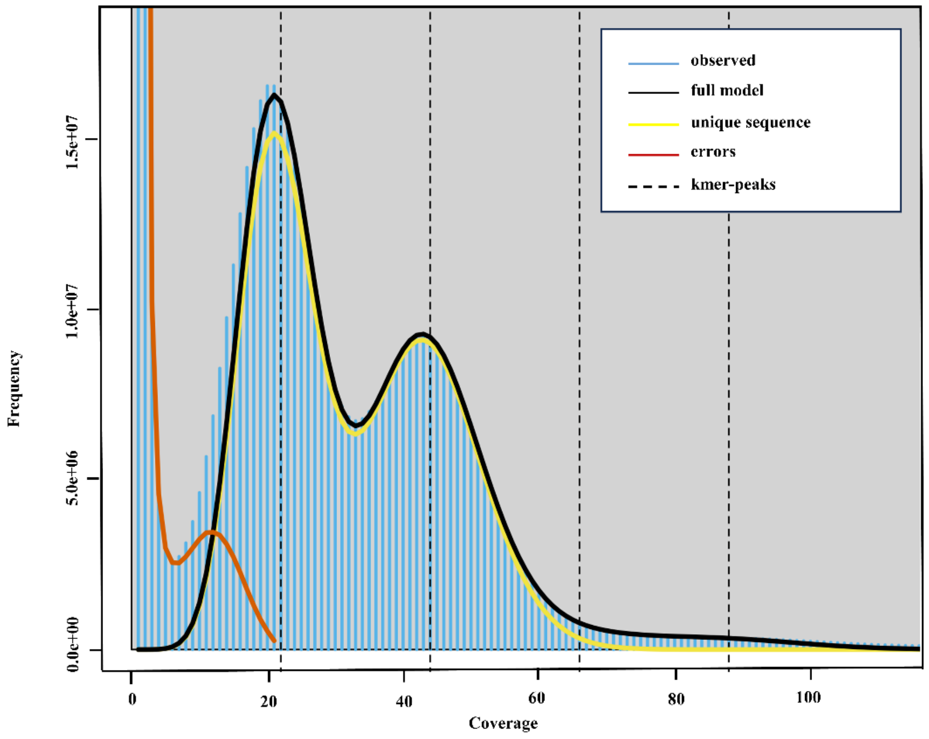

Figure S5 Estimation of *A. odorata* genome size and heterozygosity using *k-mer* size of 21.

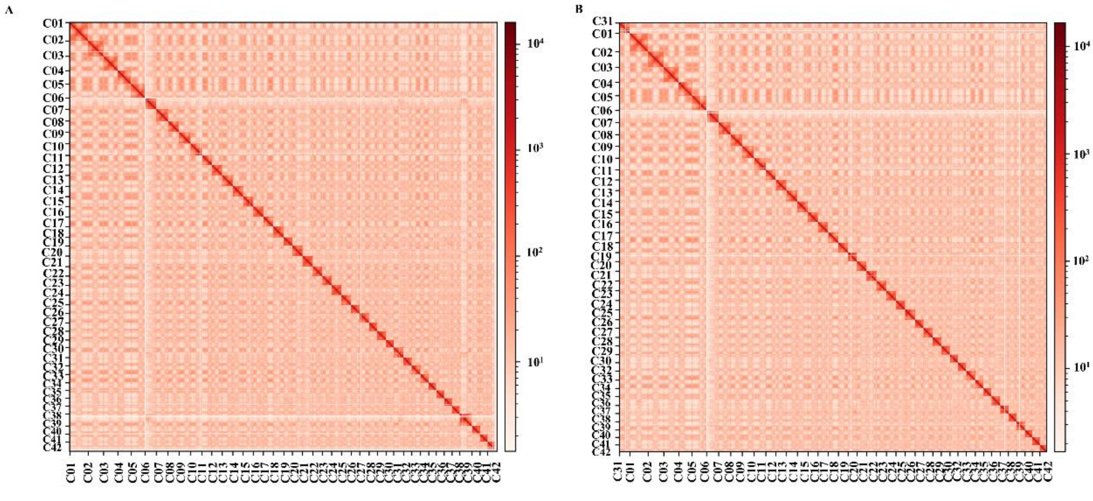

Figure S6 The Hi-C heatmap of *A. odorata.*

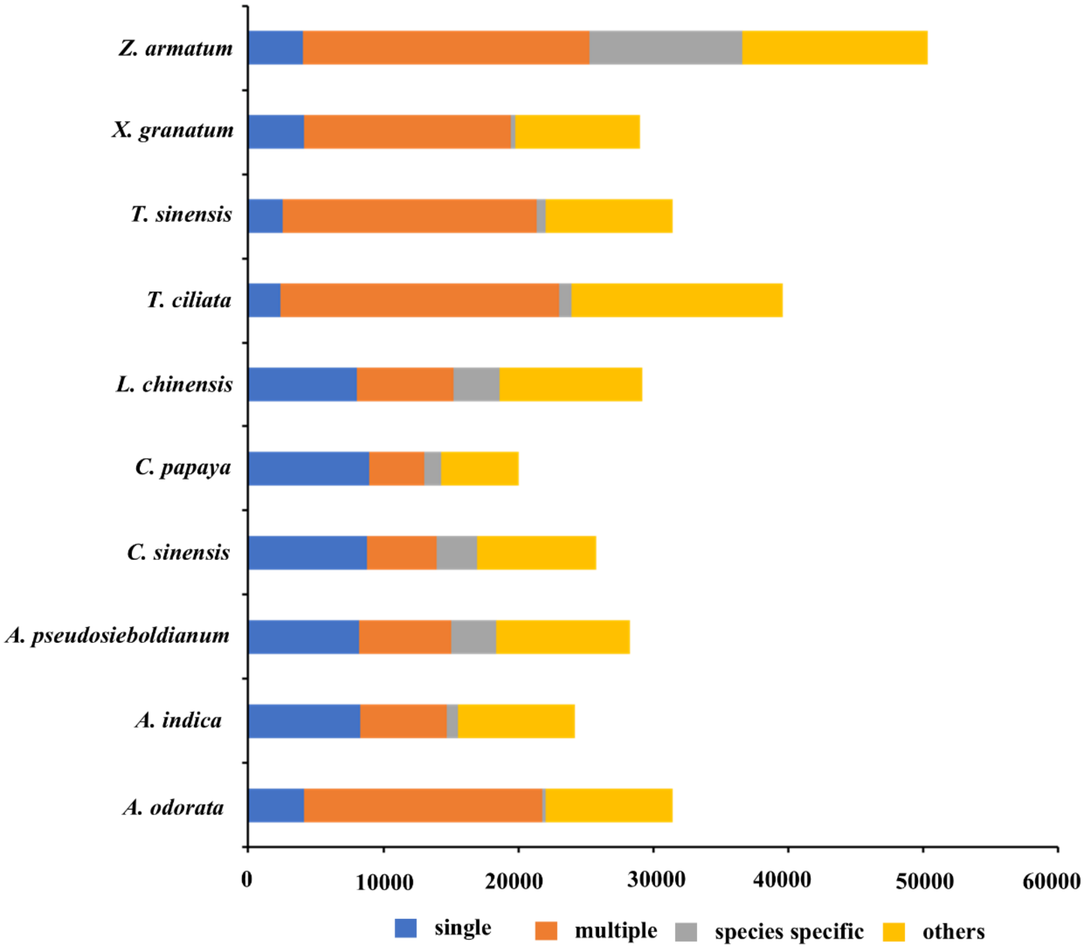

Figure S7 Gene family counts across *A. odorata* and 8 other Sapindales species and the outgroup species *C. papya*.

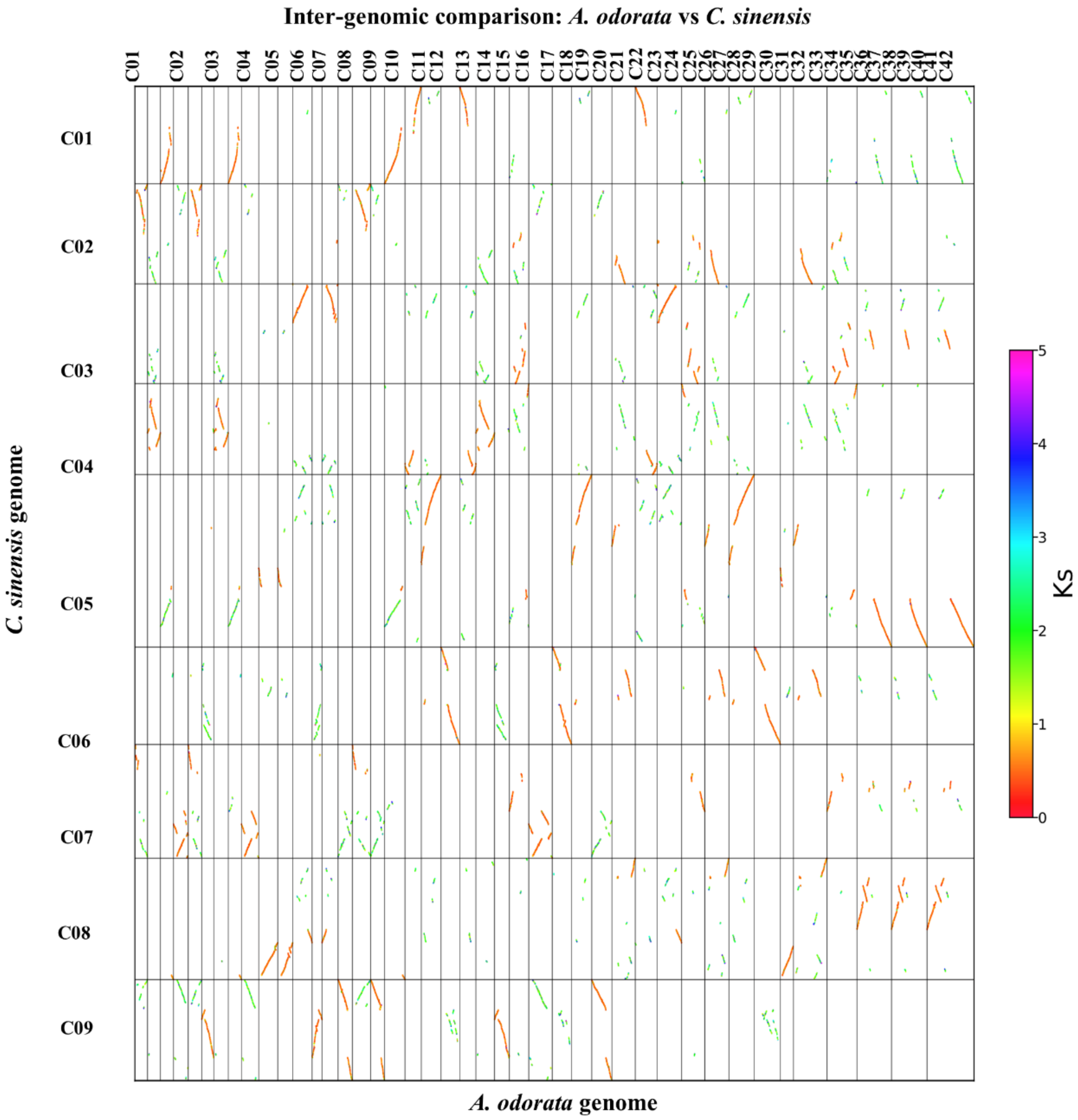

Figure S8 Dot plots syntenic blocks showing 1:3 chromosomal relationship between *C. sinensis* and *A. odorata* genome.

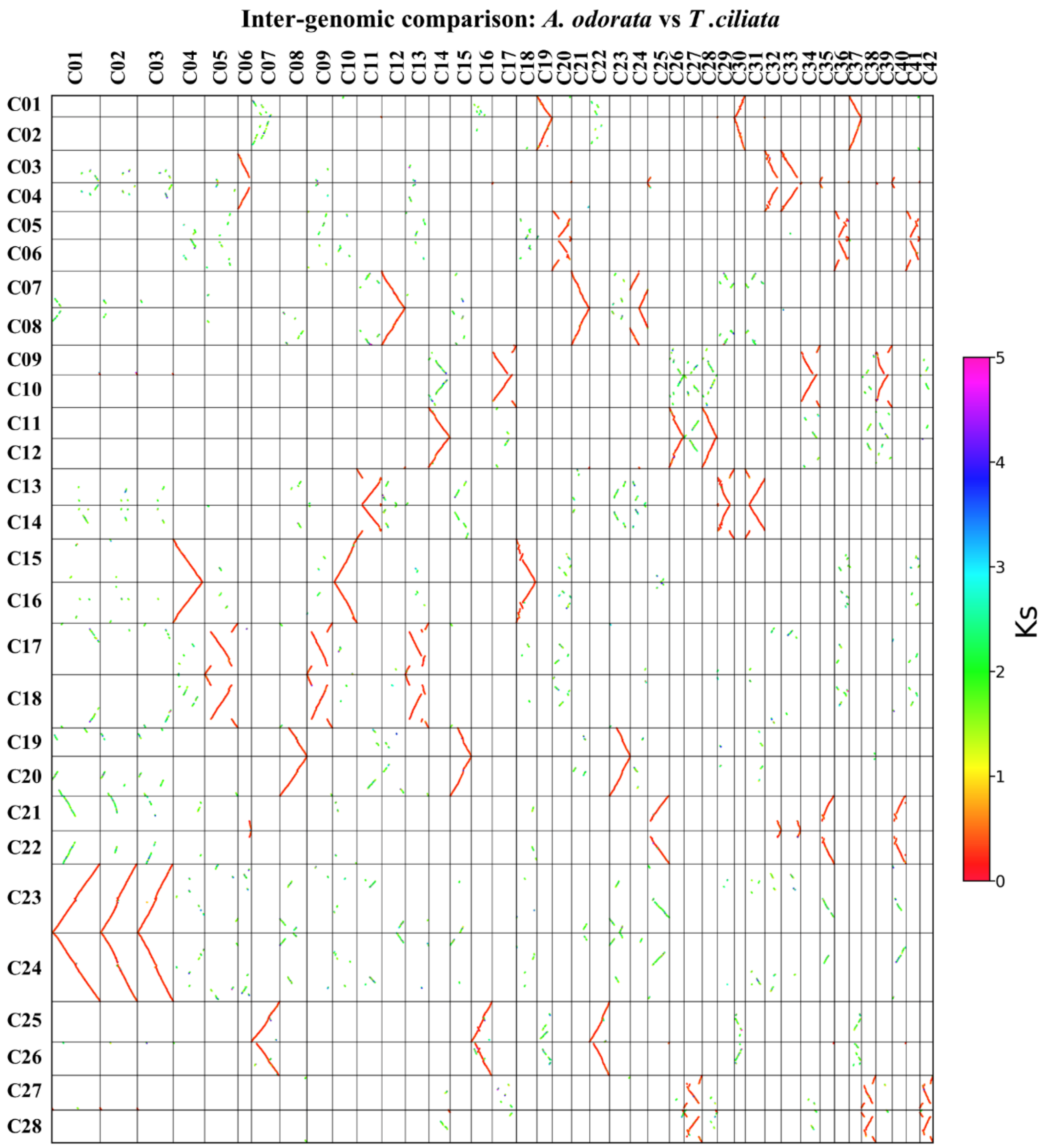

Figure S9 Dot plots syntenic blocks showing 2:3 chromosomal relationship between *T. cilita* and *A. odorata* genome.

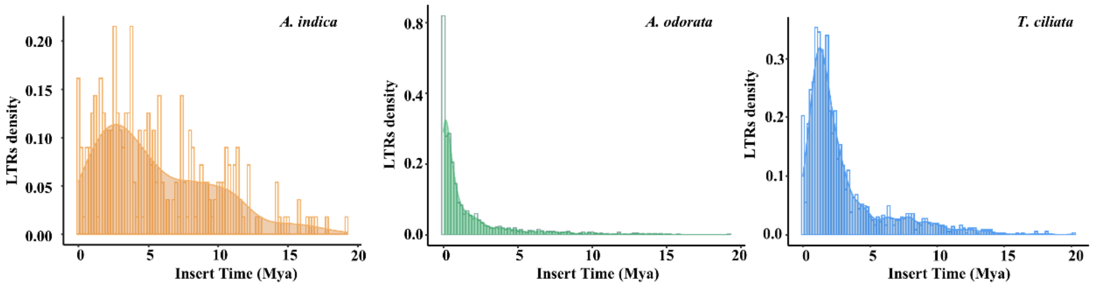

Figure S10 Histogram of LTR insertion times in *A. indica, A. odorata, and T. ciliata*.

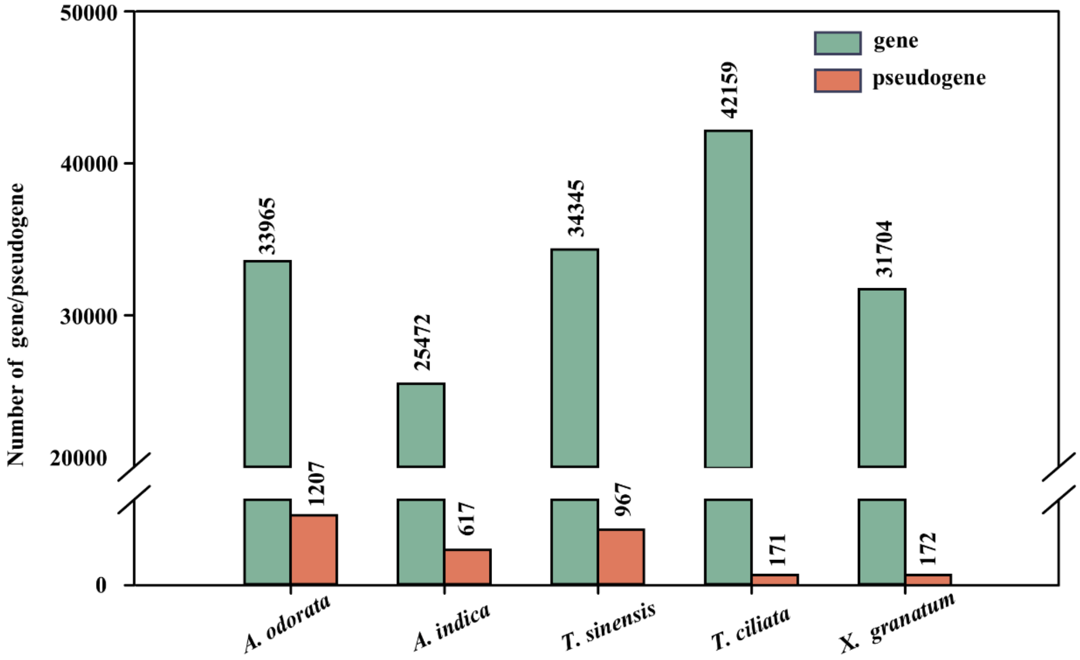

Figure S11 Genes and pseudogenes counts in five Meliaceae species.

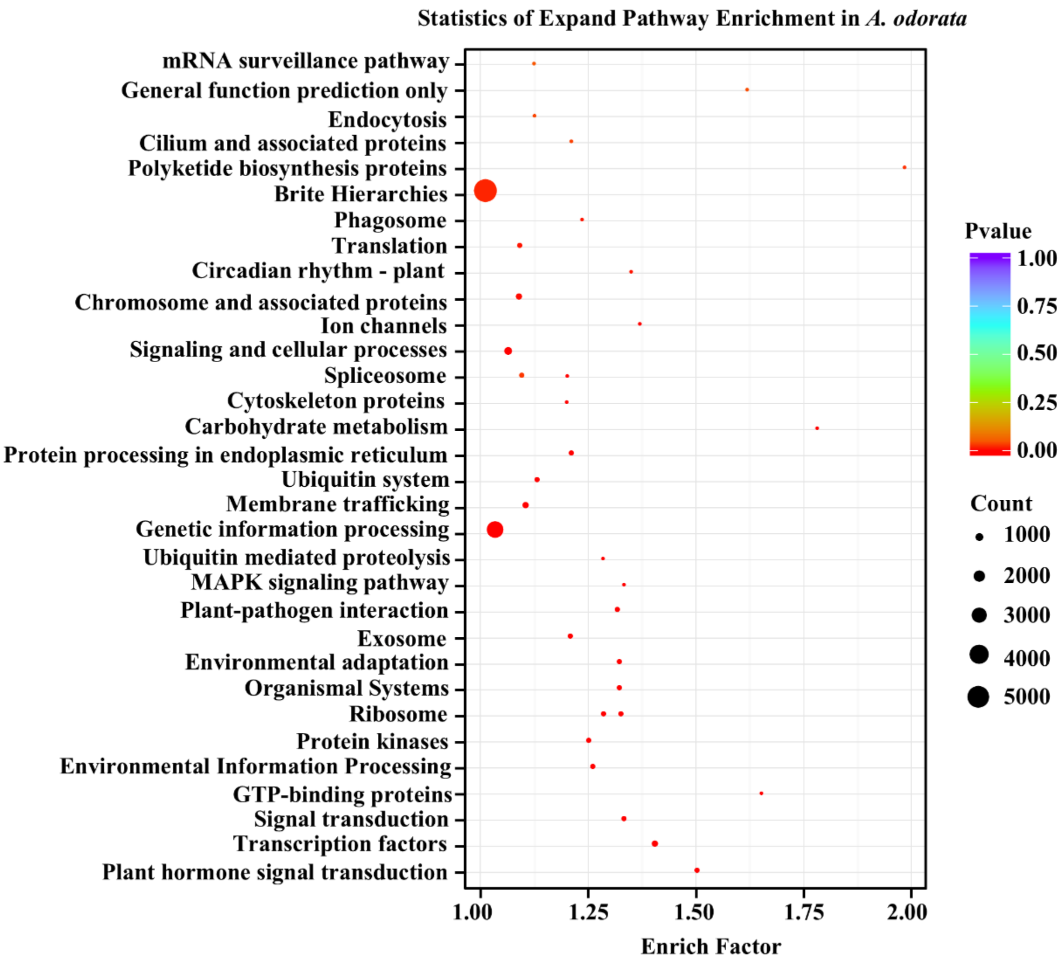

Figure S12 KEGG enrichment of expanded gene families in *A. odorata*.

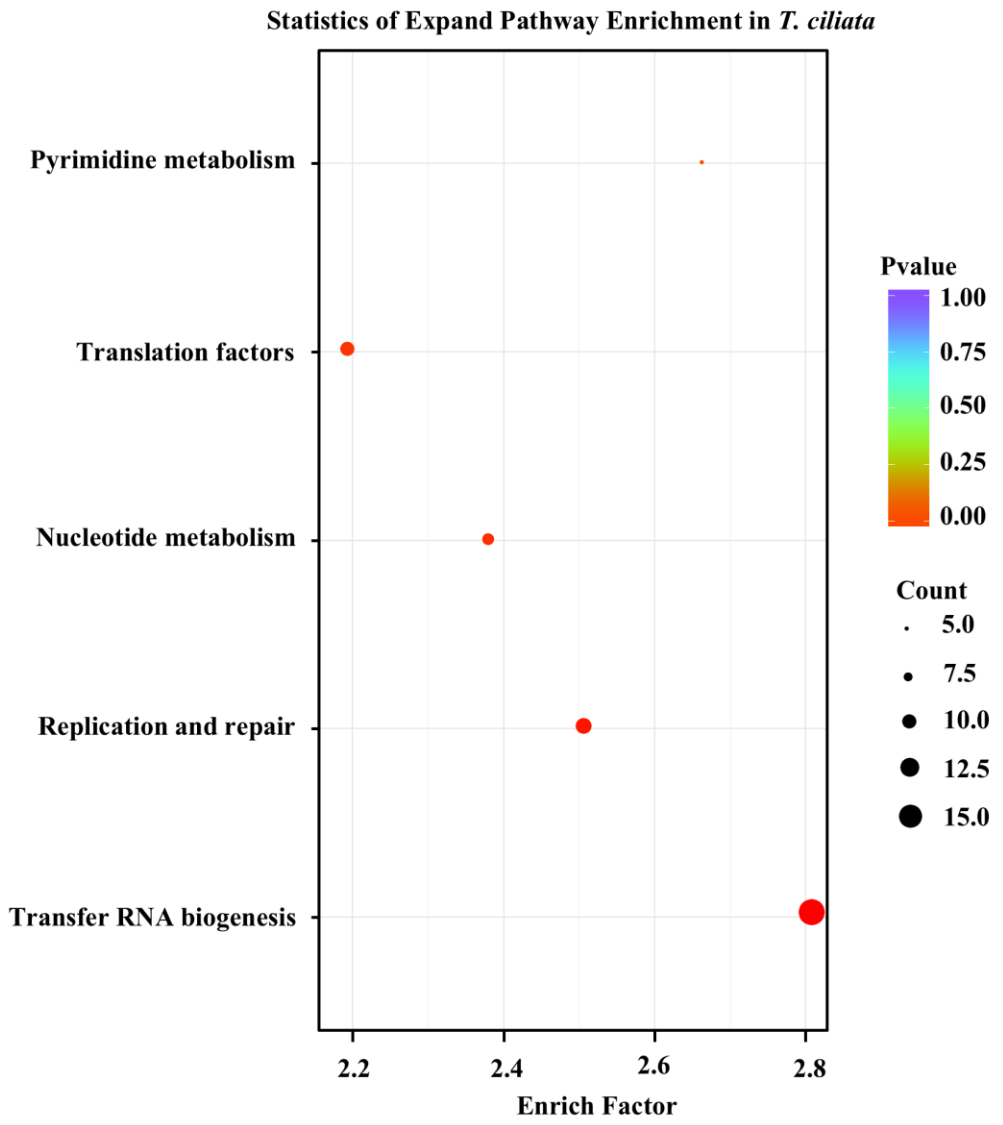

Figure S13 KEGG enrichment of expanded gene families in *T. ciliata*.

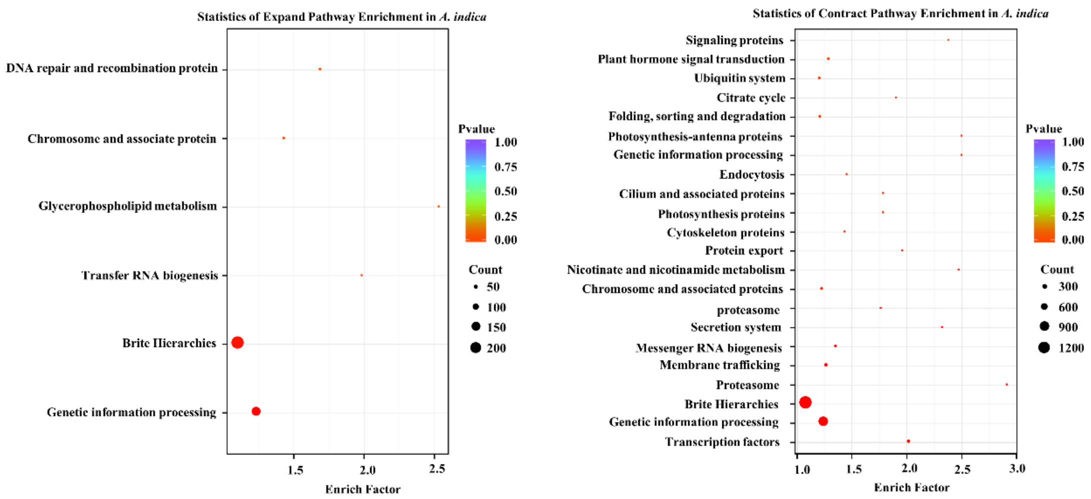

Figure S14 KEGG enrichment of expanded and contracted gene families in *A. indica.*

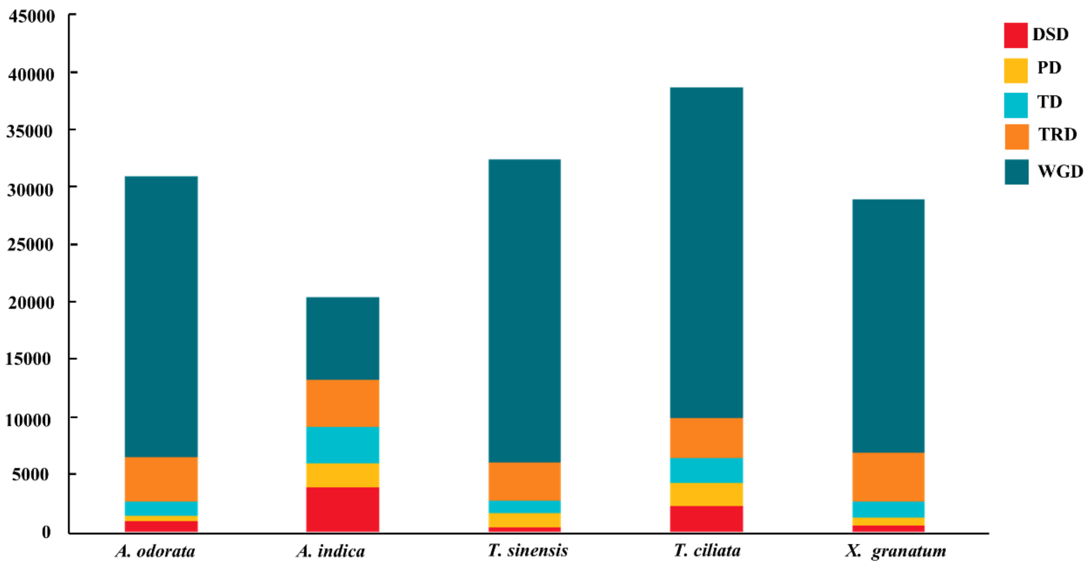

Figure S15 Categories and numbers of duplicated genes in five Meliaceae species.

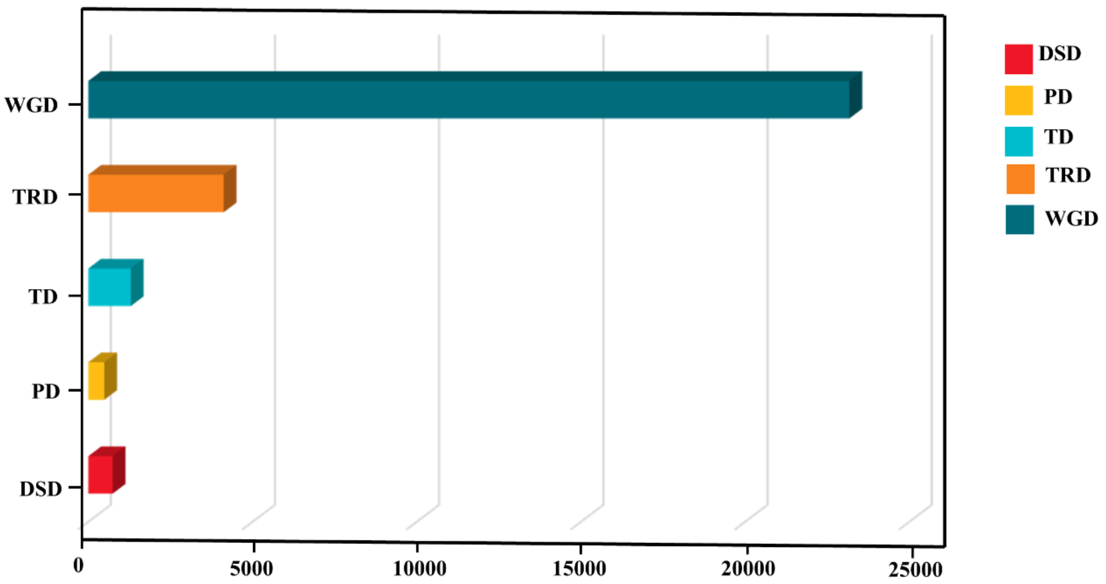

Figure S16 Categories and numbers of duplicated genes in *A. odorata*.

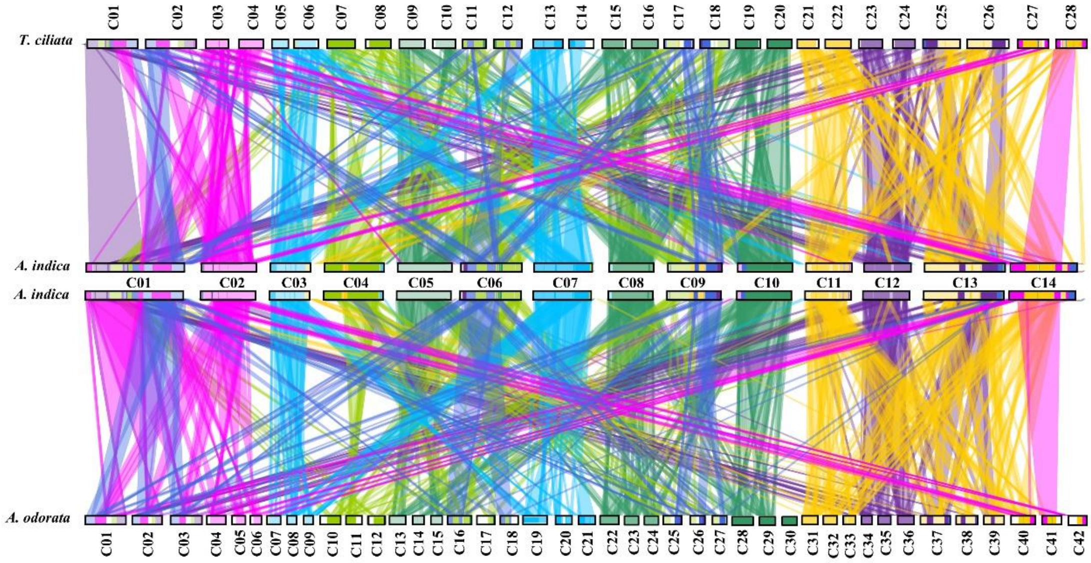

Figure S17 Segmental collinearity among *A. odorata*, *T. ciliata*, and *A. indica* based on ACEK blocks (A1-A7, B1-B7, C1-C7).

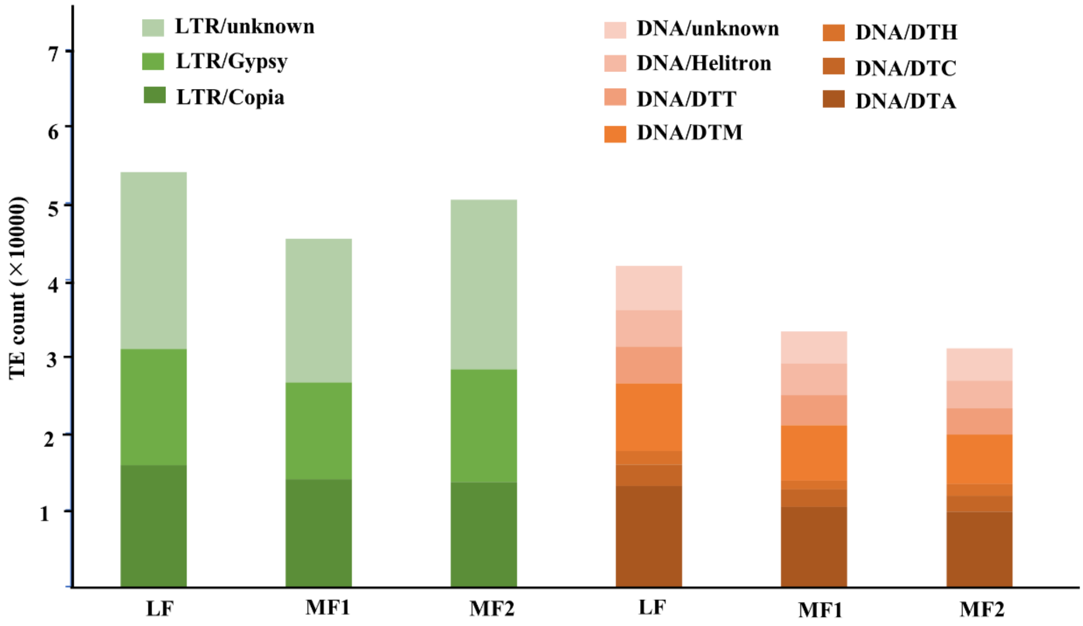

Figure S18 Categories and numbers of LTR and DNA elements in the three *A. odorata* subgenomes.

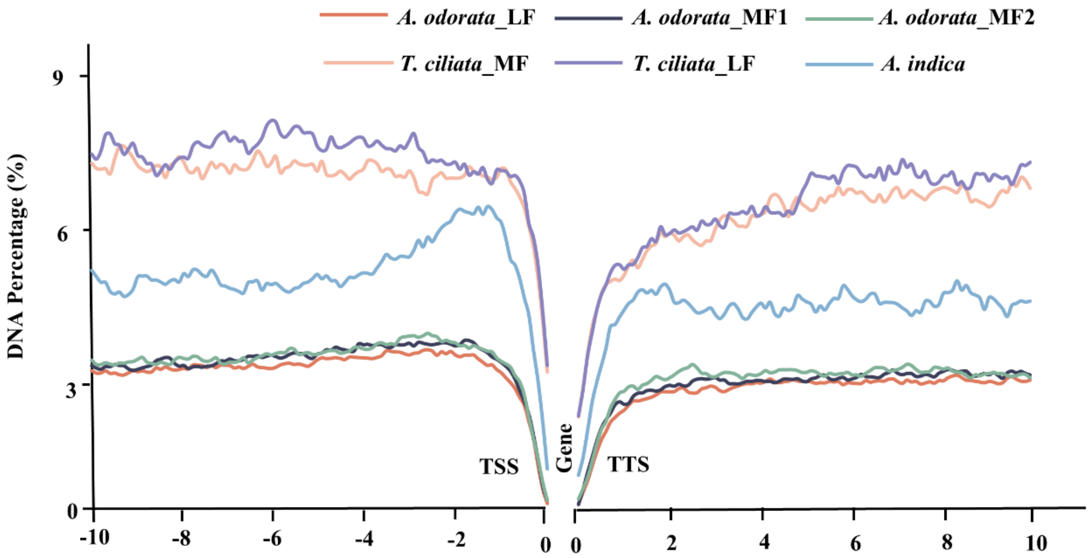

Figure S19 DNA density within 10 kb upstream and downstream of genes in *A. odorat*a and *T. ciliata* subgenomes.

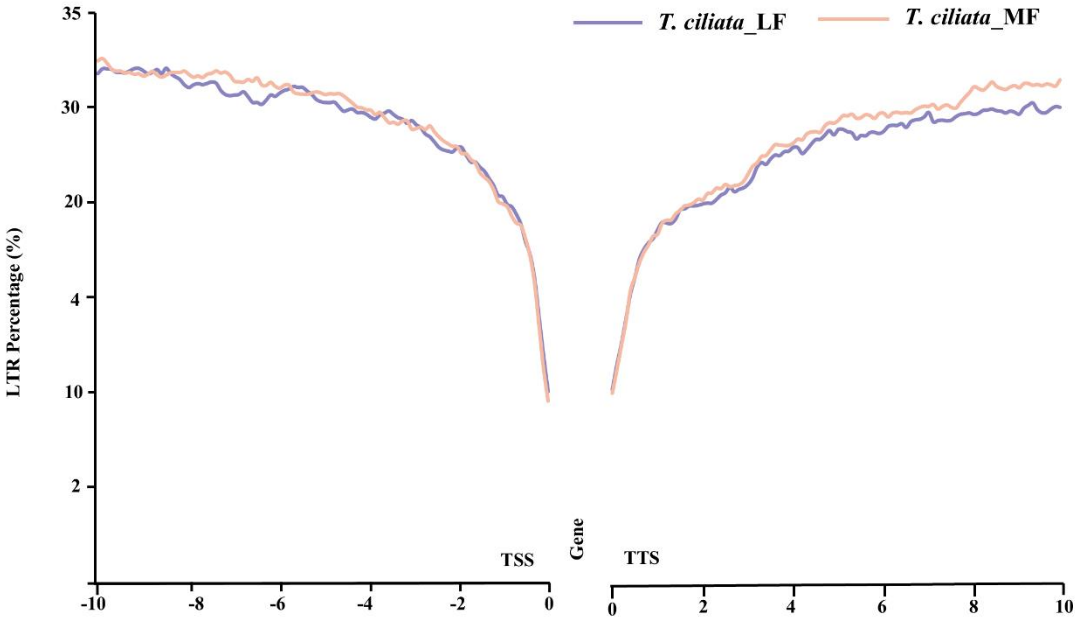

Figure S20 LTR density within 10 kb upstream and downstream of genes in *T. ciliata* subgenomes.

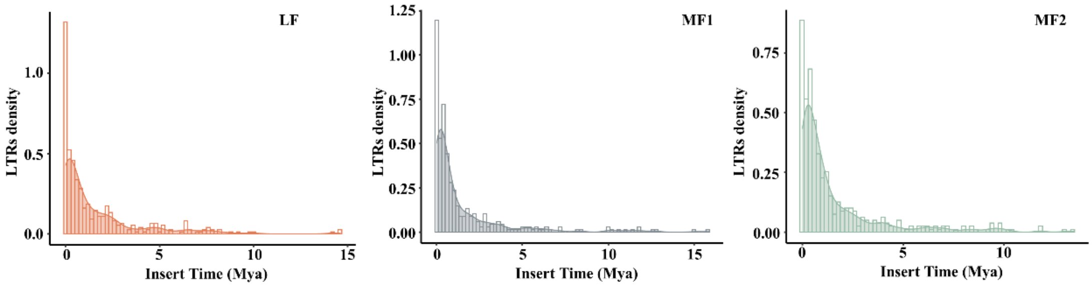

Figure S21 Histogram of LTR insertion times in the LF, MF1, and MF2 subgenomes of *A. odorata*.

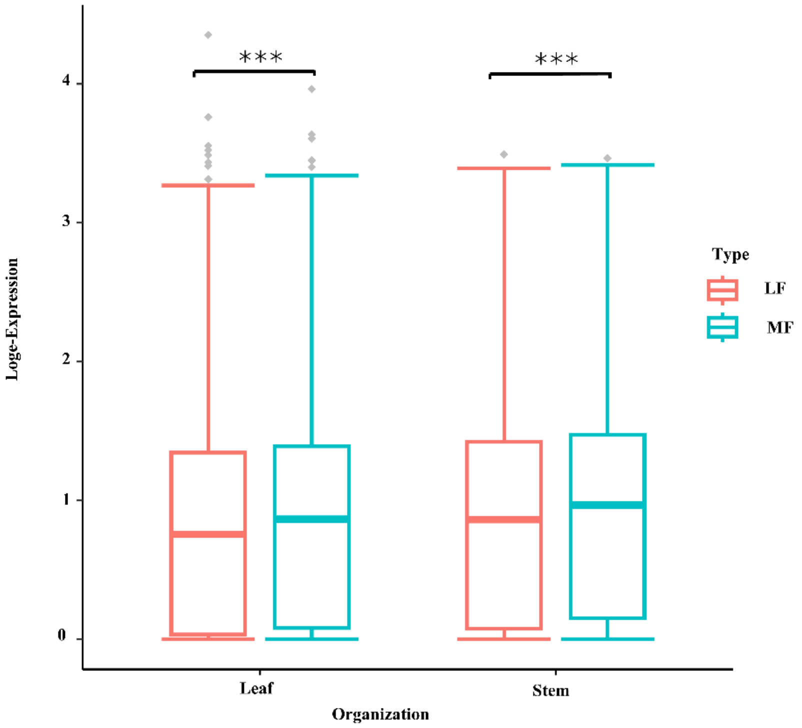

Figure S22 Box plots of homologous gene expression in leaves and stems of *T. ciliata* subgenomes.

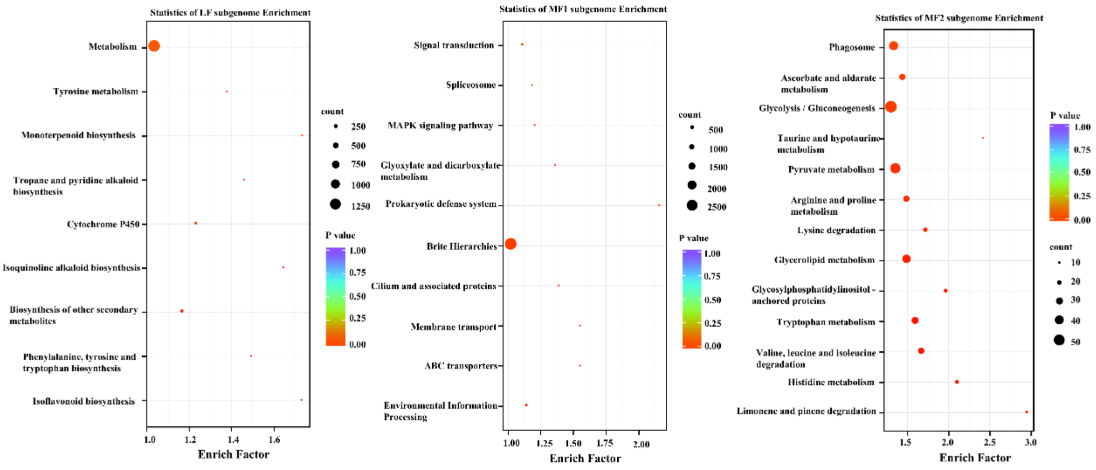

Figure S23 KEGG pathway enrichment analysis of three subgenomes gene families in *A. odorata*.

MVA pathway

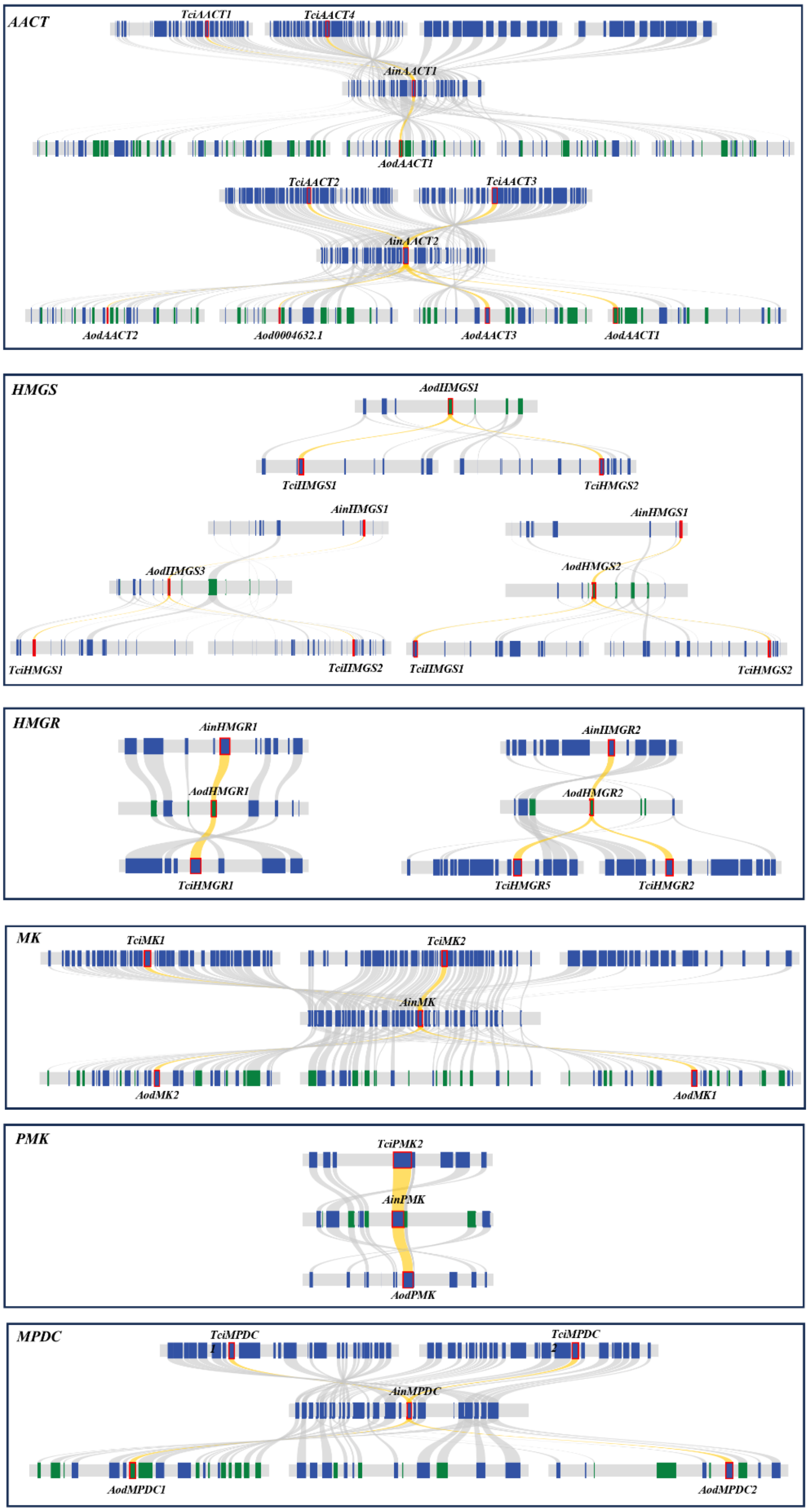

Figure S24 Synteny of key genes in the MVA pathway for terpenoid biosynthesis in *A. indica*, *T. ciliata*, and *A. odorata*.

MEP pathway

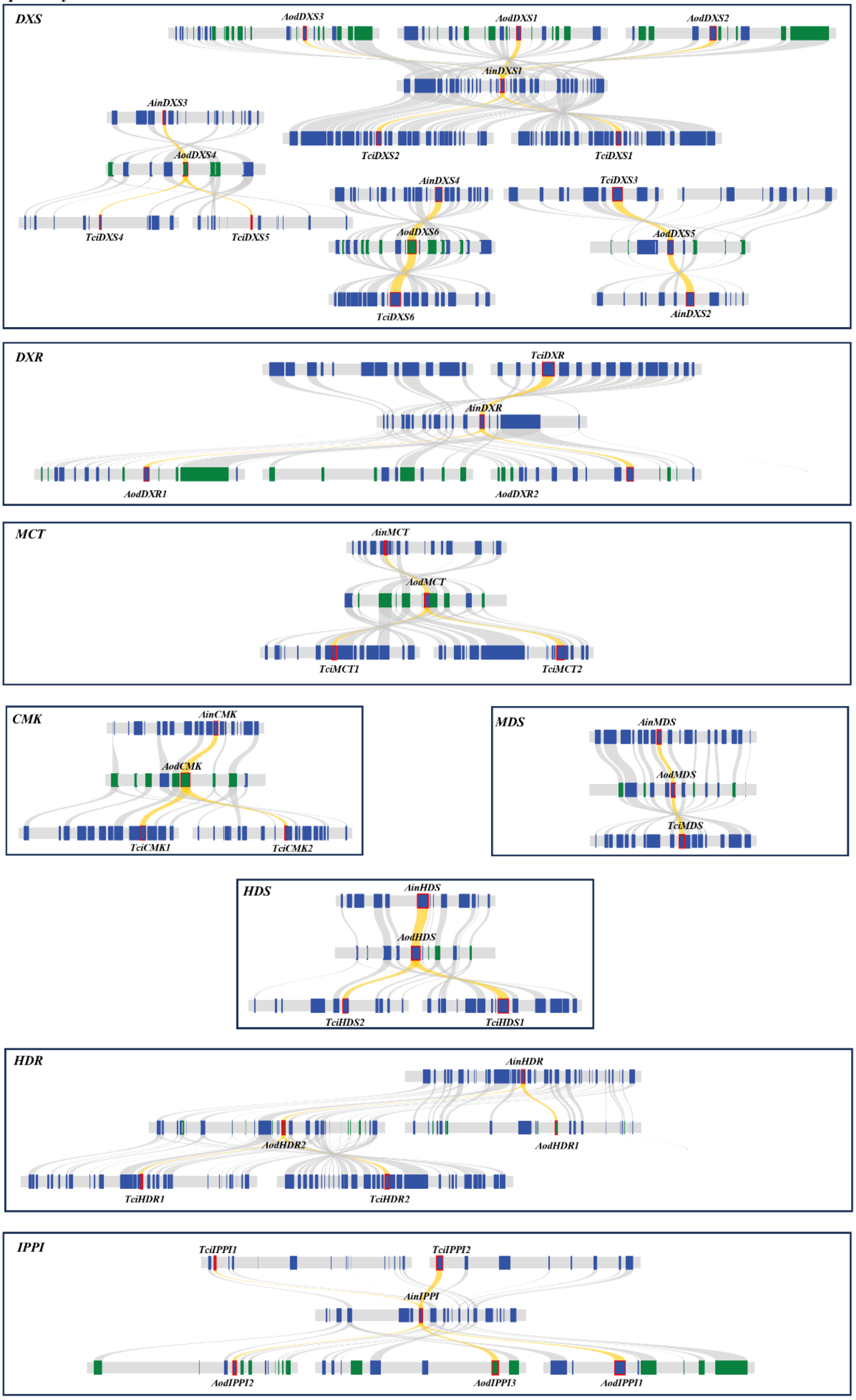

Figure S25 Synteny of key genes in the MEP pathway for terpenoid biosynthesis in *A. indica*, *T. ciliata*, and *A. odorata*.

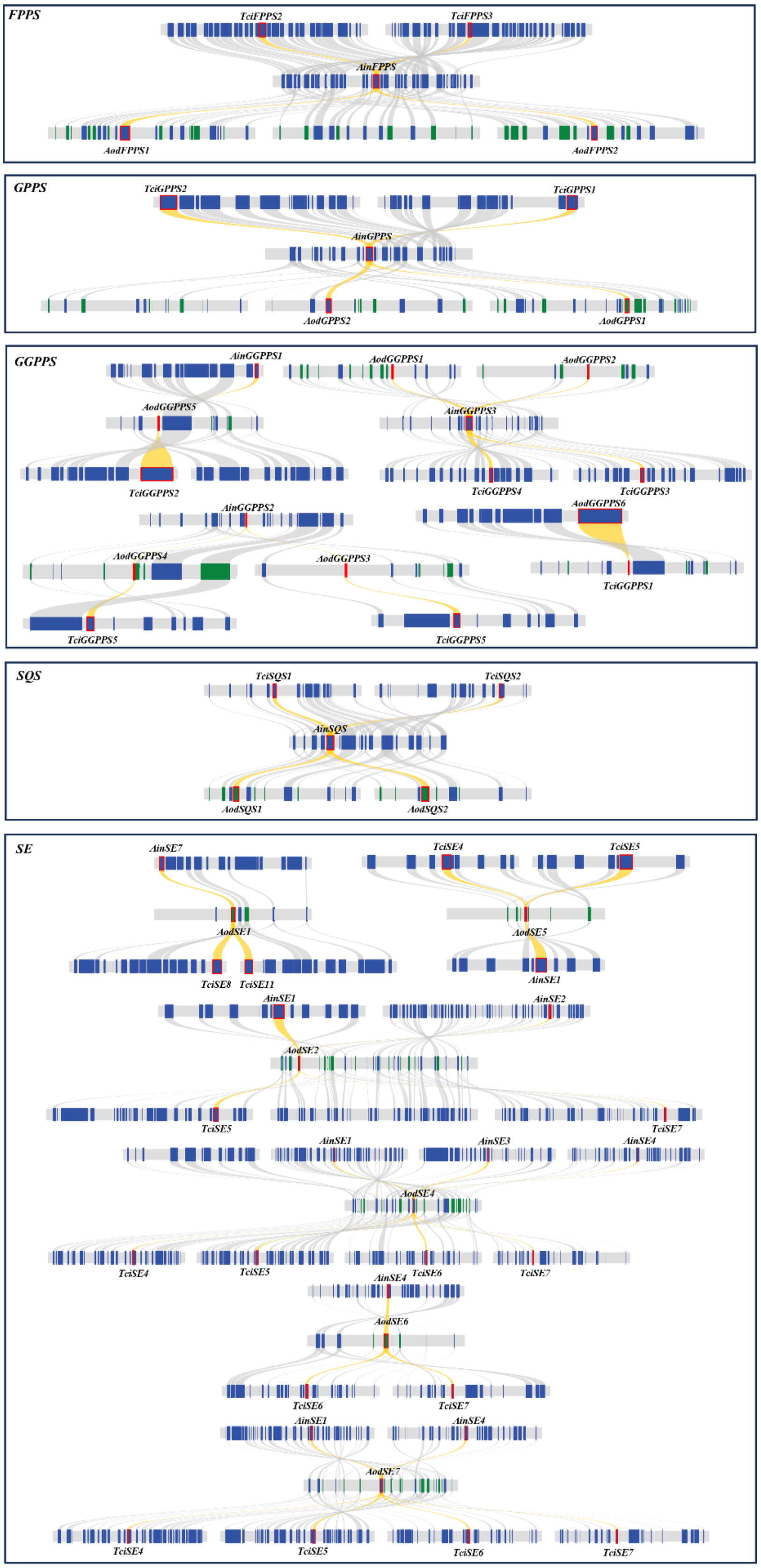

Figure S26 Synteny of key genes for terpenoid precursor biosynthesis in *A. indica*, *T. ciliata*, and *A. odorata*.

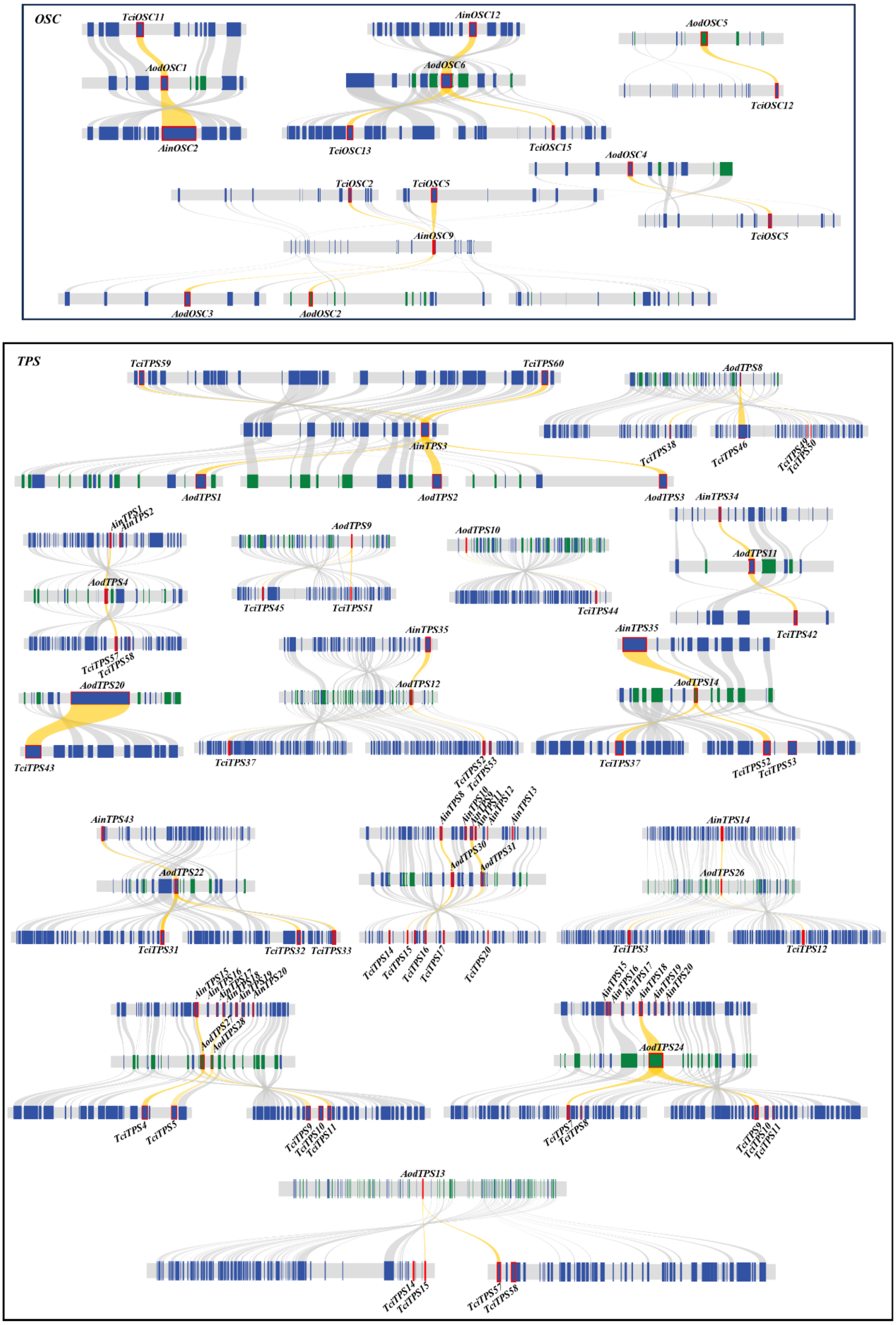

Figure S27 Synteny of key genes for terpenoid skeleton biosynthesis in *A. indica*, *T. ciliata*, and *A. odorata*.

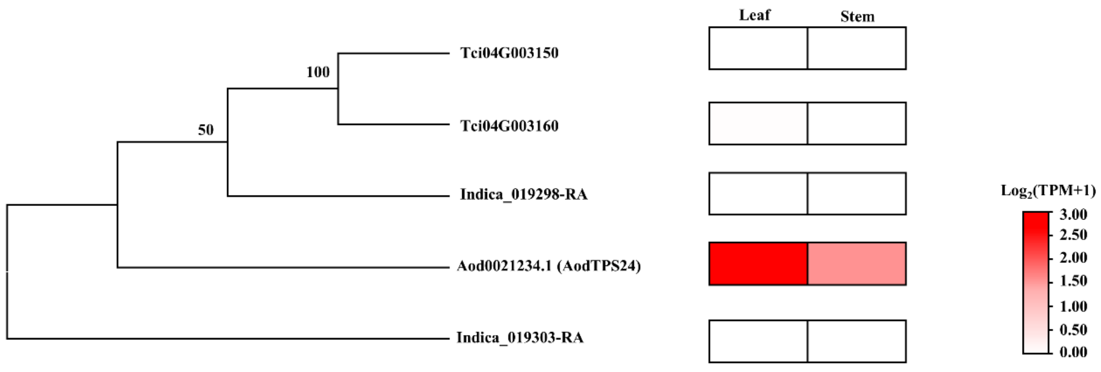

Figure S28 Heatmap of *AodTPS24* and homologous gene expression.

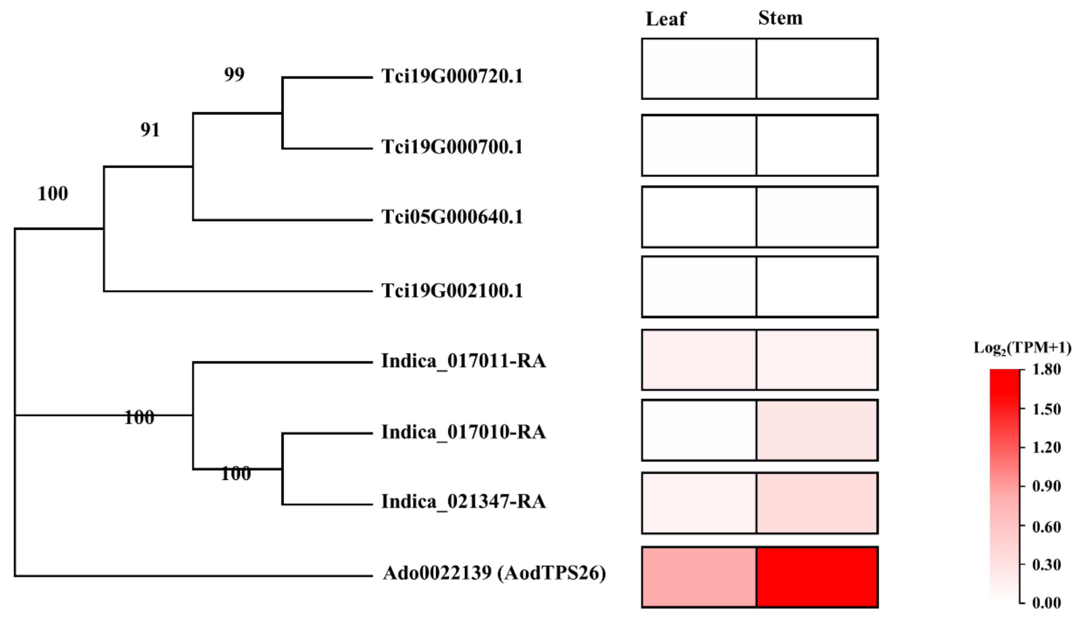

Figure S29 Heatmap of *AodTPS26* and homologous gene expression.

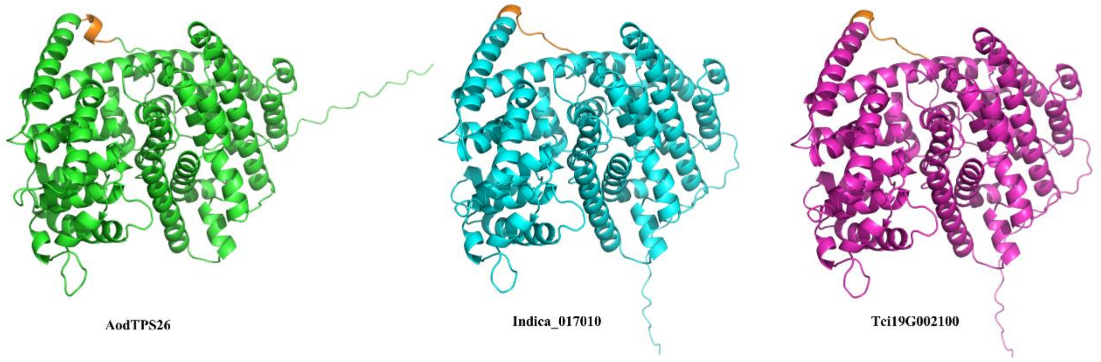

Figure S30 Predicted three-dimensional structures of AodTPS26, Indica_017010, and Tci19G002100.

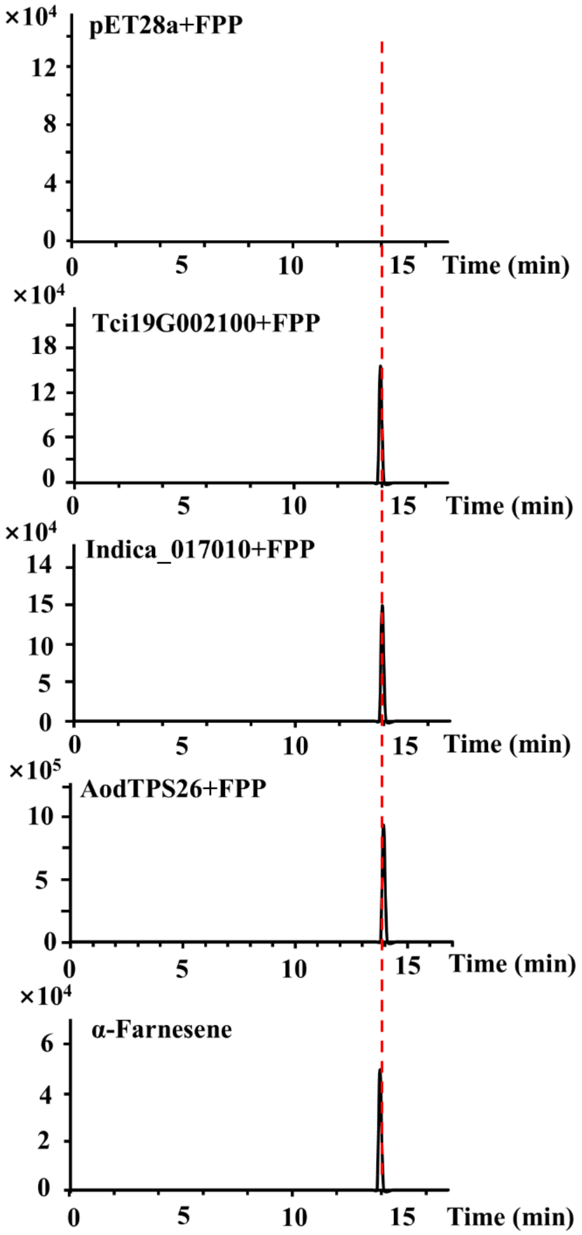

Figure S31 GC-MS spectra of volatile terpenes from overexpressed *AodTPS26*, *Tci19G002100*, and *Indica_017010*.

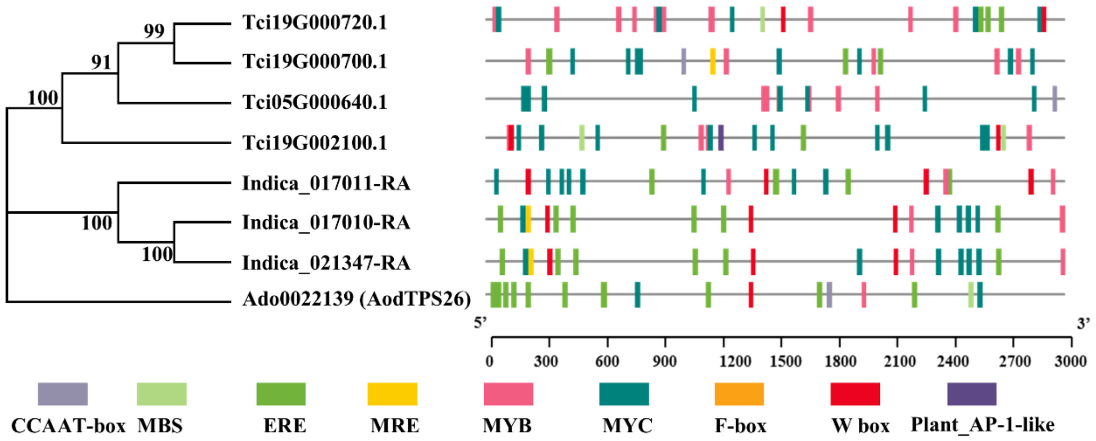

Figure S32 Cis-element analysis of the 3000 bp promoter regions of *AodTPS26* and its homologs.

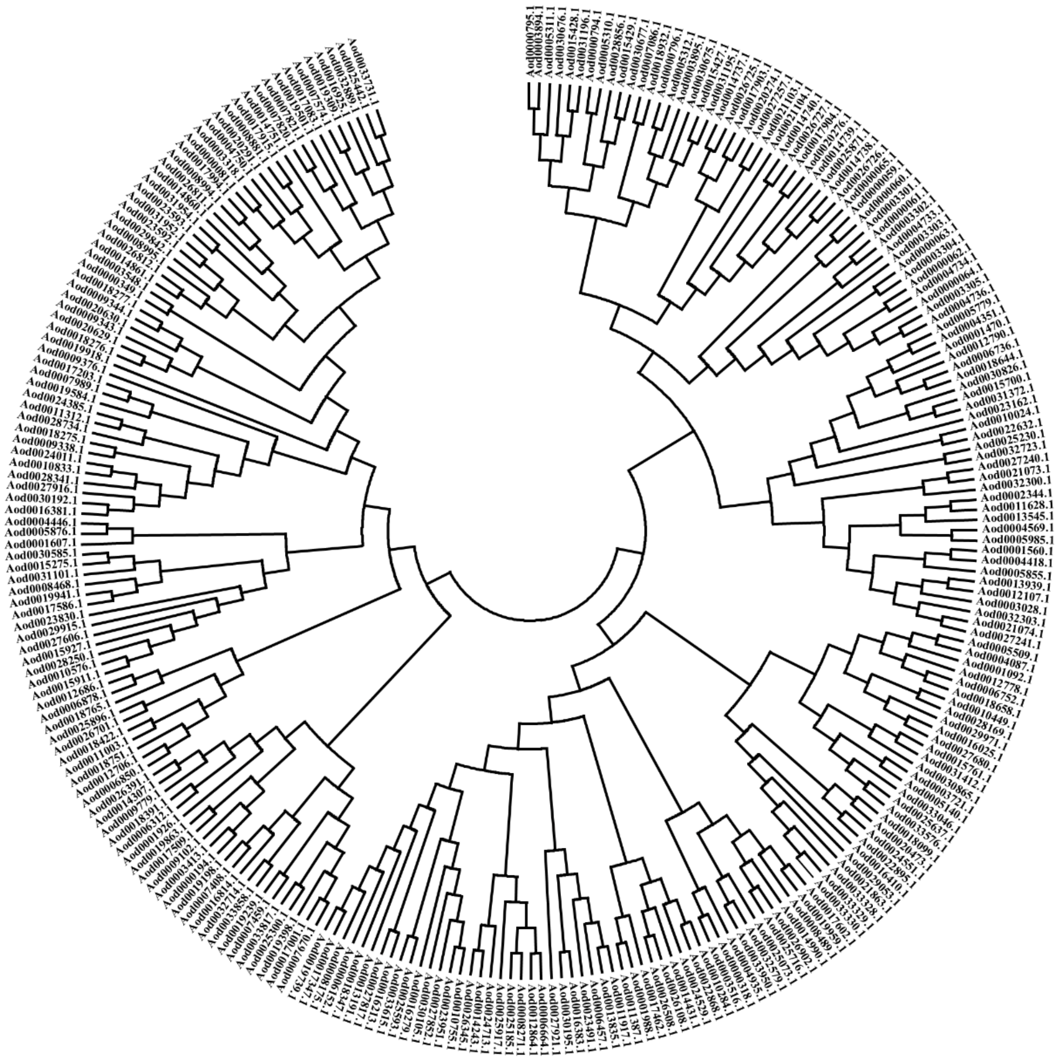

Figure S33 The phylogenetic tree of ERF.

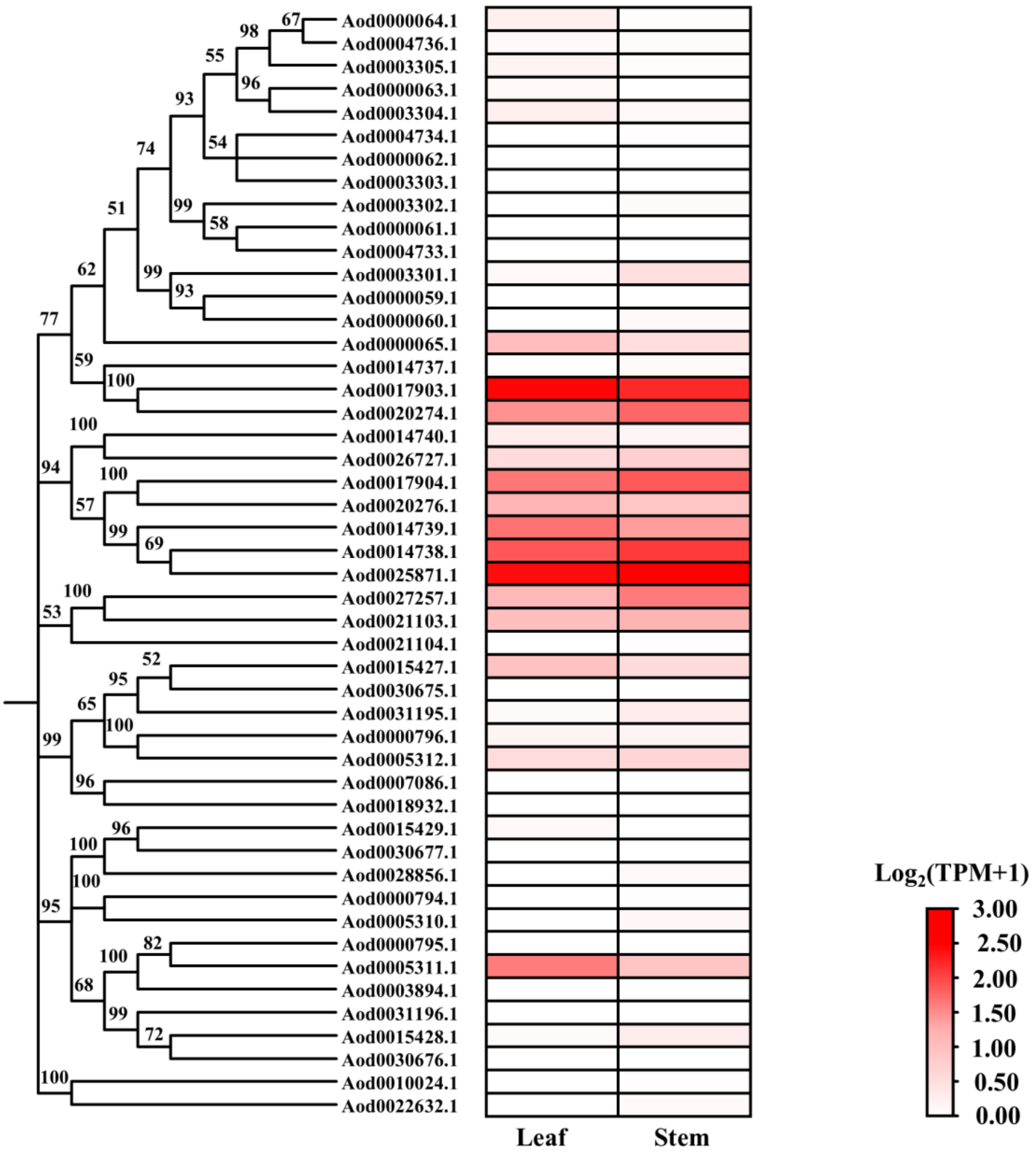

Figure S34 Heatmap of *ERF* subfamily B3 gene expression in *A. odorata* leaves and stems.
