# Supplementary material for: High-quality genome assembly of Aglaia odorata reveals evolution, terpenes diversity and abundance in Meliaceae
Source: Mol Hortic. 2026 Apr 9;6:29. doi: 10.1186/s43897-025-00212-9 (PMC13063475; doi:10.1186/s43897-025-00212-9)
Supplement: Supplementary file 2 — Supplementary Material 2: Table S1 The genome sequencing data of A. odorata. Table S2 Summary statistics of genome assembly of A. odorata. Table S3 Pseudochromosome length statistics in A. odorata genome. Table S4 Genome BUSCO assessment for A. odorata. Table S5 Transposable elements content in Hap1 of the A. odorata genome. Table S6 Transposable elements content in Hap2 of the A. odorata genome. Table S7 Functional annotations of A. odorata genes. Table S8 Statistics of non-coding RNAs in the A. odorata genome. Table S9 Protein-level BUSCO assessment for A. odorata. Table S10 Comparison of published chromosome-level Meliaceae genomes. Table S11 Resequencing mapping rate for five individuals of A. indica, T. ciliata and A. odorata. Table S12 Gene retention ratios in subgenomes of A. odorata and T. ciliata based on ACEK blocks comparing with A. indica. [file 43897_2025_212_MOESM2_ESM.pdf]

Table S1 The genome sequencing data of *A. odorata*

**Sequencing**	***A. odorata***
**Nanopore Sequencing**	
Bases (bp)	56,837,034,417
Reads number	2,728,641
Reads average length (bp)	20,868
**Hifi Sequencing**	
Bases (bp)	96,500,144,882
Reads number	4,944,094
Reads average length (bp)	19,518
**Illumina Sequencing**	
Reads (Mb)	104.09
Base (G)	31.23
Q20 (%)	97.98
Q30 (%)	94.37
GC content (%)	33.18
**Hic sequencing**	
Reads (Mb)	184.30
Base (G)	55.29
Q20 (%)	98.27
Q30 (%)	95.9
GC content (%)	38.41

Table S2 Summary statistics of genome assembly of *A. odorata*

**Genome**	**Hap1**<br>**Contig Length (bp)**	**Hap2**<br>**Contig Length (bp)**
**Primary assembly**		
N50	10705455	10455770
N90	8106949	8323957
Longest	19946120	19179946
Total	499901975	456138034
**Chromosome-level genome assembly**		
N50	12098207	10428530
N90	9719513	8393858
Longest	19933472	19179591
Total	462315564	448909388

Table S3 Pseudochromosome length statistics in *A. odorata* genome

Group	Hap1	Hap2
Chr01	19933472 bp	19179591 bp
Chr02	16593229 bp	16381681 bp
Chr03	15521094 bp	15753500 bp
Chr04	14309669 bp	14026741 bp
Chr05	15121574 bp	15305906 bp
Chr06	12228841 bp	12638374 bp
Chr07	12679647 bp	12721294 bp
Chr08	12006257 bp	12131983 bp
Chr09	12376183 bp	12479039 bp
Chr10	12452944 bp	12300182 bp
Chr11	10567270 bp	10428530 bp
Chr12	11161369 bp	11112245 bp
Chr13	11590802 bp	11501842 bp
Chr14	11163139 bp	11261607 bp
Chr15	10953710 bp	10480870 bp
Chr16	10881638 bp	10870626 bp
Chr17	10703393 bp	10635004 bp
Chr18	11512624 bp	10157642 bp
Chr19	9210109 bp	9371084 bp
Chr20	10935336 bp	10176094 bp
Chr21	11295576 bp	10292386 bp
Chr22	10249905 bp	10317125 bp
Chr23	9965419 bp	10087989 bp
Chr24	10588848 bp	9763107 bp
Chr25	10557742 bp	10381878 bp
Chr26	9185638 bp	8883306 bp
Chr27	10231334 bp	9685589 bp
Chr28	8945616 bp	8909238 bp
Chr29	9458428 bp	9211041 bp
Chr30	8106709 bp	8323948 bp
Chr31	10839428 bp	10842223 bp
Chr32	8725063 bp	8811832 bp
Chr33	9475172 bp	9370544 bp
Chr34	8874826 bp	8809761 bp
Chr35	8237972 bp	8261645 bp
Chr36	8575426 bp	9494773 bp
Chr37	8456872 bp	8393858 bp
Chr38	8340874 bp	8618990 bp
Chr39	13207912 bp	8796244 bp
Chr40	8807682 bp	7596267 bp
Chr41	8210953 bp	7987423 bp
Chr42	10075869 bp	7156386 bp

Table S4 Genome BUSCO assessment for *A. odorata*

Items	Hap1		Hap2	
	Number	Percentage (%)	Number	Percentage (%)
Complete BUSCOs	1587	98.3	1583	98.1
Complete and single-copy BUSCOs	1309	81.1	1309	81.1
Complete and duplicated BUSCOs	278	17.2	274	17.0
Fragmented BUSCOs	18	1.1	22	1.4
Missing BUSCOs	9	0.6	9	0.5
Total lineage BUSCOs	1614	/	1614	/

Table S5 Transposable elements content in Hap1 of the *A. odorata* genome

**Class**	**Super family**	**Count**	**Masked (bp)**	**Masked (%)**
DNA_transposon		18333	4653680	1.01%
LTR				
	Copia	56929	42111779	9.11%
	Gypsy	55912	34519002	7.47%
	unknown	79543	52492748	11.35%
TIR				
	CACTA	8840	2268005	0.49%
	Mutator	30210	10222902	2.21%
	PIF_Harbinger	9970	3353072	0.73%
	Tc1_Mariner	15522	3408924	0.74%
	hAT	46269	14083549	3.05%
	polinton	43	29490	0.01%
low_complexity		117	433484	0.09%
nonLTR				
	L1_LINE	38	18956	0.00%
	LINE_element	1225	1047077	0.23%
	Penelope	177	48744	0.01%
	tRNA_SINE	464	188650	0.04%
	unknown	598	298930	0.06%
nonTIR				
	helitron	15993	4582849	0.99%
repeat_region		6979	2055278	0.44%
Total		347162	175817119	38.03%

Table S6 Transposable elements content in Hap2 of the *A. odorata* genome

Class	Super family	Count	Masked (bp)	Masked (%)
DNA_transposon		17559	4536213	1.01%
LTR				
	Copia	56588	41223007	9.18%
	Gypsy	52167	33350376	7.43%
	unknown	71153	49812761	11.10%
TIR				
	CACTA	828	299869	0.07%
	Mutator	16715	4523795	1.01%
	PIF_Harbinger	4463	1217656	0.27%
	Tc1_Mariner	13630	3208192	0.71%
	hAT	51944	15042663	3.35%
	unknown	3	327	0.00%
low_complexity		108	428834	0.10%
nonLTR				
	I_LINE	6	3877	0.00%
	L1_LINE	84	35449	0.01%
	LINE_element	1723	859404	0.19%
	Penelope	546	209349	0.05%
	tRNA_SINE	260	111909	0.02%
	unknown	324	219055	0.05%
nonTIR				
	helitron	15873	3945033	0.88%
repeat_region		13440	4127610	0.92%
retrotransposon		70	25992	0.01%
Total		317484	163181371	36.35%

Table S7 Functional annotations of *A. odorata* genes

**Database**	**Hap1**		**Hap2**	
	Number	Percentage (%)	Number	Percentage (%)
Swissprot	23,854	70.23	23,854	70.42
KEGG	4,560	13.43	4,560	13.46
KOG	18,250	53.73	18,250	53.88
GO	928	2.73	928	2.74
NR	33,160	97.63	33,160	97.89
Total annotated	33,178	97.68	33,178	97.95

Table S8 Statistics of non-coding RNAs in the *A. odorata* genome

**Items**	**Hap1**<br>**Number**	**Hap2**<br>**Number**
rRNA	212	355
tRNA	409	405
miRNA	93	94
snoRNA	1397	1135

Table S9 Protein-level BUSCO assessment for *A. odorata*

**Items**	**Hap1**		**Hap2**	
	Number	Percentage (%)	Number	Percentage (%)
Complete BUSCOs	1582	98.1%	1578	97.8%
Complete and single-copy BUSCOs	1352	83.8%	1348	83.5%
Complete and duplicated BUSCOs	230	14.3%	230	14.3%
Fragmented BUSCOs	8	0.5%	10	0.6%
Missing BUSCOs	24	1.4%	26	1.6%
Total lineage BUSCOs	1614	/	1614	/

Table S10 Comparison of published chromosome-level Meliaceae genomes

Species	*A. odorata* (this study)	*A. indica*	*T. ciliata*	*T. sinensis*	*X. granatum*
Sequencing platform	Illumina, ONT, Hifi, Hic	Illumina, PacBio, Hic	Illumina, ONT, Hic	Illumina, ONT, Hic	Illumina, PacBio, Hic
Genome size (Mb)	462.32	281	520.64	596.35	300.08
Contig N50 (Mb)	10.94	6	4.33	1.53	9.56
Number 0f genes	33965	25767	42159	24245	31705
Total repeat element (Mb)	175.82	94.38	291.67	385.22	124.58
Repeat element ratio (%)	38.03	34.56	56.02	64.56	41.52
PseudoChromosome number	42	14	28	28	28

Table S11 Resequencing mapping rate for five individuals of *A. indica*, *T. ciliata* and *A. odorata*

Species	*A. odorata* (%)	*T. ciliata* (%)	*A. indica* (%)
Individual 1	95.93	99.03	89.29
Individual 2	96.10	98.90	90.16
Individual 3	96.36	99.51	91.97
Individual 4	96.88	99.31	74.95
Individual 5	96.47	99.48	85.30

Table S12 retention ratios in subgenomes of *A. odorata* and *T. ciliata* based on ACEK blocks comparing with *A. indica*

	Retention rate in *A. odorata*/ *A. indica*			Retention rate in *T. ciliata* / *A. indica*	
	LF	MF1	MF2	LF	MF
A1	0.49	0.36	0.34	0.83	0.77
B1	0.58	0.41	0.40	0.85	0.81
C1	0.59	0.43	0.37	0.79	0.68
A2	0.41	0.34	0.32	0.67	0.63
B2	1.01	0.25	0.13	0.50	0.49
C2	0.27	0.22	0.17	0.79	0.74
A3	1.10	0.37	0.35	0.77	0.76
B3	1.73	1.00	0.83	2.57	2.48
C3	1.14	0.48	0.41	0.95	0.54
A4	0.58	0.46	0.34	1.12	0.79
B4	0.71	0.29	0.24	0.50	0.47
C4	0.82	0.55	0.50	0.93	0.55
A5	0.51	0.28	0.27	0.84	0.47
B5	1.59	0.45	0.07	1.28	1.27
C5	0.40	0.35	0.27	0.36	0.35
A6	3.16	0.45	0.41	0.95	0.87
B6	0.47	0.34	0.32	0.87	0.77
C6	1.76	0.29	0.21	0.94	0.88
A7	0.77	0.67	0.57	0.87	0.78
B7	0.63	0.52	0.48	0.50	0.47
C7	1.28	0.31	0.24	0.66	0.65
